# Desorption of positive and negative ions from activated field emitters at atmospheric pressure

**DOI:** 10.1177/14690667221133388

**Published:** 2022-10-18

**Authors:** Jürgen H. Gross

**Affiliations:** Institute of Organic Chemistry, Heidelberg University, Heidelberg, Germany

**Keywords:** Field desorption, atmospheric pressure ionization, electrospray ionization (ESI), activated field emitter, Fourier transform-ion cyclotron resonance, accurate mass, ionic liquid, polymers, liquid injection field desorption/ionization (LIFDI), negative ions, ionization process, desorption ionization

## Abstract

Field desorption (FD) traditionally is an ionization technique in mass spectrometry (MS) that is performed in high vacuum. So far only two studies have explored FD at atmospheric pressure or even superatmospheric pressure, respectively. This work pursues ion desorption from 13-µm activated tungsten emitters at atmospheric pressure. The emitters are positioned in front of the atmospheric pressure interface of a Fourier transform-ion cyclotron resonance (FT-ICR) mass spectrometer and the entrance electrode of the interface is set to 3–5 kV with respect to the emitter. Under these conditions positive, and for the first time, negative ion desorption is achieved. In either polarity, atmospheric pressure field desorption (APFD) is robust and spectra are reproducible. Both singly charged positive and negative ions formed by these processes are characterized by accurate mass-based formula assignments and in part by tandem mass spectrometry. The compounds analyzed include the ionic liquids trihexyl(tetradecyl) phosphonium tris(pentafluoroethyl) trifluorophosphate) and 1-butyl-1-methylpyrrolidinium bis(trifluoromethylsulfonyl)imide, the acidic compounds perfluorononanoic acid and polyethylene glycol diacid, as well as two amino-terminated polypropylene glycols. Some surface mobility on the emitter is prerequisite for ion desorption to occur. While ionic liquids inherently provide this mobility, the desorption of ions from solid analytes requires the assistance of a liquid matrix, e.g. glycerol.

## Introduction

The present works explores ion desorption in a transition zone located within a triangle between (*i*) classical field desorption (FD), (*ii*) nano-electrospray ionization (nanoESI), and (*iii*) – to some degree – ambient desorption/ionization (ADI). In order to contextualize this study, these three pillars shall briefly be characterized:

i) Field ionization (FI) and FD – both performed in high vacuum – are known for decades as soft ionization techniques generally delivering intact positive molecular ions, M^+•^, or adduct ions like [M + H]^+^ and [M + alkali]^+^ of neutral molecular compounds.^1–4^ Field ionization requires the highest electric field strength to effect tunneling of an electron from the neutral analyte molecule towards the emitter.^1,5,6^
Field desorption of ions does occur at field strengths being about a hundred times lower.^5,7,8^ Thus, ionic compounds are perfectly suitable for analysis by FD because the intact cations C^+^, often accompanied by cluster ions [C_n+1_A_n_]^+^, are easily desorbed, i.e. FD does necessarily require FI as an ionization process for ion desorption to occur as long as there are preformed ions available on the emitter surface. The implementation of FD as liquid-injection field desorption/ionization (LIFDI)^9–15^ additionally offers sample application to the emitter under the complete exclusion of moisture and air.^4,12,13,16–18^ Whatever the setup may be, FI, FD, and LIFDI are traditionally performed in high vacuum where ion formation and ion desorption are effected by very strong electric fields. To locally enhance the electric fields to 1–2 V Å^–1^ as required for the FI pathway of M^+•^ ion formation to occur, the field emitters are activated by growing microneedles on their surface.^19–21^ The indene-based activation of tungsten wire serves for the production of commercially available emitters.^20,21^ While being extremely fragile, such activated tungsten wire emitters provide thousands of field-enhancing microneedles and a large surface for deposition of a sample layer.
ii) Electrospray ionization relies on electric fields to electrophoretically separate ions in solution and to cause electrolytic solutions to form a continuous spray of highly charged microdroplets.^22,23^ For the most part, ESI is performed using capillaries of 50–100 µm inner diameter, a flow of analyte solution of 5–500 µl min^–1^, and spray voltages between sample capillary and counter electrode of 2–4 kV.^24–26^ Flows of just 20–100 nl min^–1^ are realized by nanoESI, where the liquid flow from a capillary through a narrow exit of several µm in diameter is effected by the joint action of capillary and electrostatic forces due to the high voltage applied.^27–29^ A simplified and potentially more robust variant of nanoESI based on (disposable) copper and platinum wire loop or wire coil emitters, respectively, has been introduced^30–33^ where sample solutions are applied to the surface of these wires rather than inside a capillary. In particular noteworthy is a type of ESI emitters based on tungsten oxide nanowires (TON).^34^ The surfaces of these TON emitters for ESI show some resemblance to activated field emitters.
iii) The development of desorption electrospray ionization (DESI)^35^ and direct analysis in real time (DART)^36,37^ initiated the exploration of dozens of ambient desorption/ionization (ADI) techniques.^37–40^

These exciting developments have opened our eyes for the fact that almost anything that can generate some ions at atmospheric pressure and that can be placed in front of an atmospheric pressure ionization (API) interface of a suitable mass spectrometer may serve as some sort of ion source for mass spectrometry (MS). Examples in this field include ESI-derived techniques like paper spray ionization,^41^ tissue paper-assisted spray ionization,^42^ field-induced wooden tip ESI,^43^ the use of a sharp stainless steel needle in front of an API interface,^44^ and the use of an insulating fiber as sampling probe and ionization substrate.^45^ Finally, carbon fiber ionization^46,47^ represents a technique closer related to atmospheric pressure chemical ionization (APCI).^48,49^

All of these approaches^30–34,40,42–47^ demonstrate that the surface of essentially any object providing one or numerous sharp tips can potentially act as a means of sample supply and electric field enhancement enabling ion desorption from a liquid film into the gas phase provided this sampling device is at high potential relative to a counter electrode. Typically, this counter electrode is provided by the sampling cone at the entrance of an API interface.

Obviously, modern mass spectrometry instrumentation is dominated by API interfaces. In order to adapt FD to API instrumentation while suppressing electric discharges, and thus, avoiding destruction of the emitter, FD has been performed at superatmospheric pressure (6 bar).^5^ In that study, bare 20-µm tungsten wire emitters at emitter potentials of 9–12 kV positioned at 1.6 and 5.0 mm distance to the counter electrode were employed, and moreover, the setup permitted emitter heating to be applied. Preformed positive ions of various ionic and highly polar compounds delivered high quality spectra.^5^

Another study focused on the effect of increased reaction rates in high electric fields.^6^ In this work, standard 13-µm activated tungsten emitters were used under ambient conditions. The emitters were set to 4–5 kV with respect to the counter electrode at 3–15 mm distance (orifice of API interface). Positive ions, mostly protonated molecules, [M + H]^+^, but in one instance also molecular ions, M^+•^, were observed.^6^

The present work further pursues the use of activated tungsten field emitters to effect ion desorption at atmospheric pressure and under ambient conditions. In contrast to previous work employing a linear ion trap^5^ or an Orbitrap instrument,^6^ respectively, experiments are performed using a Fourier transform-ion cyclotron resonance (FT-ICR) mass spectrometer. Moreover, encouraged by recent own and other's work,^50,51^ the formation of negative ions and the enhancement of ion desorption by a liquid matrix have been explored for the first time in atmospheric pressure field desorption (APFD).

## Experimental

### Mass spectrometer

A Bruker Apex-Qe FT-ICR mass spectrometer (Bruker Daltonics, Bremen, Germany) equipped with a 9.4 T superconducting magnet and an ESI-to-MALDI switchable Dual Source MTP was used. The instrument offers tandem MS by mass-selection of precursor ions in a linear quadrupole (Q) in front of the FT-ICR analyzer. The mass spectrometer was controlled by the Bruker ApexControl software (V 3.0.0) and data analysis was performed using the Bruker DataAnalysis software (V 4.3).

Ions were collected for 0.5–1.0 s prior to ICR mass analysis in the RF-only accumulation hexapole (h2). Ions were excited and detected using standard settings from previous work.^52–54^ External mass calibrations for positive-ion and negative-ion mode were established in ESI mode using Agilent Tune Mix (G1969-85000).^55–57^ Mass accuracy was generally in the order of 1 ppm. For tandem MS, precursor ions were selected by the quadrupole and activated by collision-induced dissociation (CID) with the argon buffer gas in h2 by applying an offset voltage.

To yield a final FT-ICR mass spectrum, normally 16 transients were accumulated. When the range *m/z* 200–2500 was selected, a 512 k data points transient resulted in a resolving power of *R* = 55,000 at *m/z* 483, an 1 M data points transient resulted in a resolving power of *R* = 110,000 at *m/z* 483, respectively.

### Atmospheric pressure FD ion source and operation

The emitter holder of a Bruker nanoESI source was replaced by a custom-built aluminum piece to clamp the FD emitter. The nanoESI source already provided an *x*,*y*,*z*-adjustable sample stage to position the emitter holder as required. Moreover, it offered observation optics by a built-in CCD camera and a monitor.

**Figure 1.**
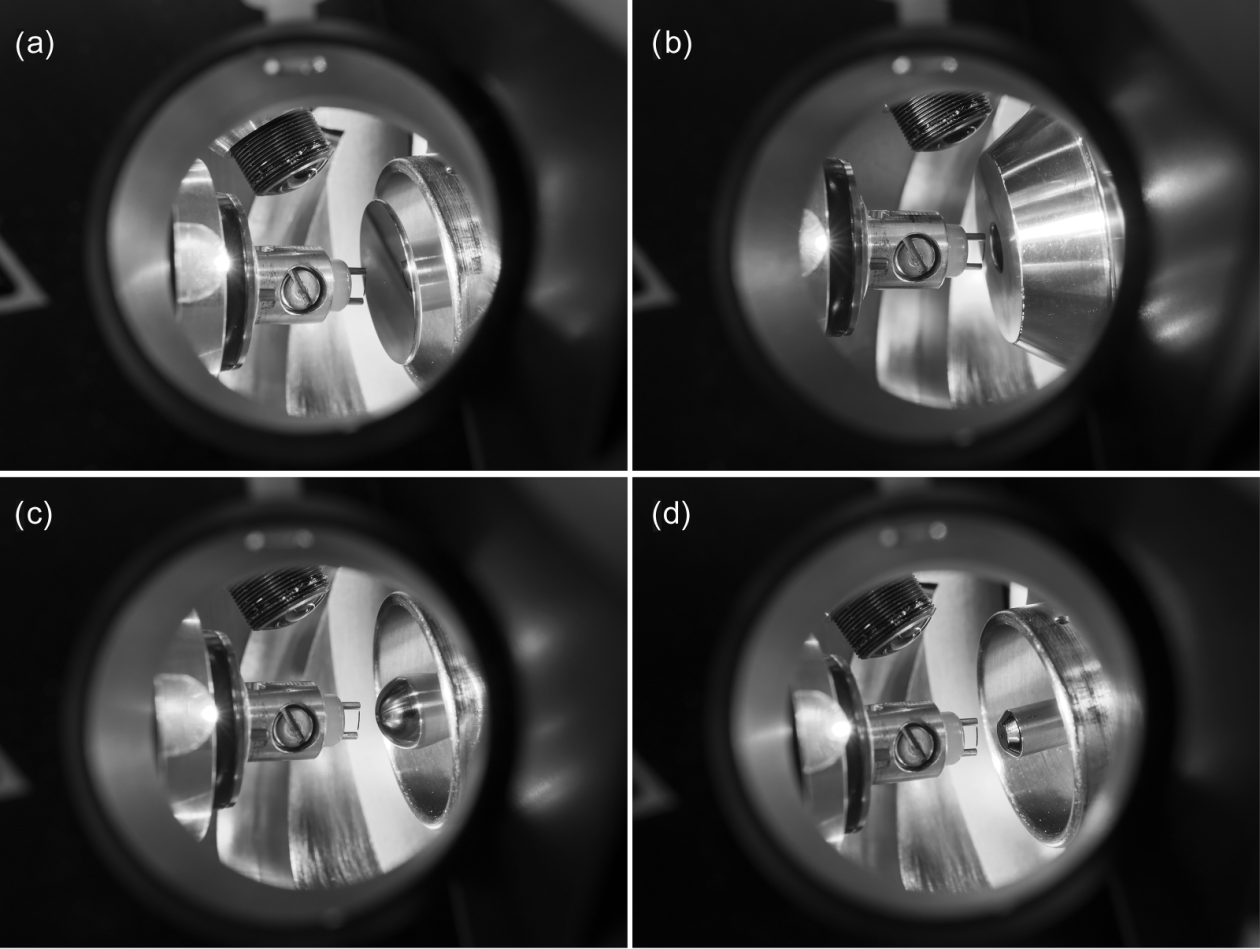
Configurations to arrange the activated emitter in front of the counter electrode provided by the Bruker API interface. Photographs were taken through the right CCD camera port after unmounting this camera. The left side CCD camera is visible at the top, the emitter holder to the left, and the respective entrance of the API interface on the right: (a) dedicated flat and polished spray shield of the nanoESI source (C1), (b) conventional ESI source entrance with complete spray shield and metal cap on the transfer capillary behind it (C2), (c) rounded cap only on the transfer capillary (C3), and (d) bare transfer capillary (C4). Either of the configurations C1 to C4 allowed ion desorption to occur.

The nanoESI source could be opened and closed via a hinge with sufficient precision as to reproducibly position an emitter for operation. The entire nanoESI source was grounded and high voltage was solely applied to the counter electrode provided by the API interface (as in all present Bruker ESI sources). Ion desorption was explored in different configurations ranging from the dedicated flat and polished spray shield (orifice 0.35 mm in diameter) of the nanoESI source (C1) over the conventional ESI source with complete spray shield and rounded metal cap (orifice 0.90 mm in diameter) on the glass transfer capillary (orifice 0.50 mm in diameter) behind it (C2), to the cap alone on the transfer capillary (C3), to bare the transfer capillary (C4, as used in DART) (Figure 1). Either of the four configurations C1 to C4 allowed ion desorption to occur. In practice, the standard ESI configuration (C2) and the bare transfer capillary (C4) worked best; C2 was least critical in terms of emitter positioning. Further photographs to explain the ion source setup and operation are provided in the Supplementary Material (Figures S1 and S2).

Activated field emitters based on 13-µm tungsten wires (Linden CMS, Weyhe, Germany) were used. The emitters were of the standard type used for the JEOL AccuTOF series of instruments.^50,58^

High voltage was adjusted via the settings provided by the API source controls. The nebulizer gas for ESI was switched off at all times, the drying gas was either off or set to 1.0 l min^–1^ at 100 °C. In C1 and C2 both capillary voltage and spray shield voltage were set, in C3 and C4 the capillary voltage was the only relevant setting. All other instrument settings were exactly as in ESI operation.

All samples were manually delivered to the emitter as solutions at 0.5–2.0 mg ml^–1^ by using a 10-µl microliter syringe while the emitter was clamped into the emitter holder. The emitter was then mounted to the nanoESI source. After the runs, the emitter was rinsed with solvent to remove excessive analyte. The same emitter could be used for several tens of acquisitions.

### Analytes

The compounds used the include ionic liquids (ILs, Merck KGaA, Darmstadt, Germany) trihexyl(tetradecyl)phosphonium tris(pentafluoroethyl)trifluorophosphate) and 1-butyl-1-methylpyrrolidinium bis(trifluoromethylsulfonyl)imide, the highly polar and acidic compounds polyethylene glycol diacid (average molecular weight 600 u, PEGDA-600) and perfluorononanoic acid (PFNA), both Sigma-Aldrich, (Steinheim, Germany), and two basic oligomers (both Huntsman (Germany) GmbH), commercially available under the trade name Jeffamine D-400 (doubly amine terminated PPG with average molecular weight of about 430 u) and Jeffamine M-2005 (singly amine terminated PPG with average molecular weight of about 2000 u). The analytes are compiled in Table 1.

**Table 1.** Compounds analyzed by APFD-MS.

Compound Name	Formula	Ion Structures
Trihexyl(tetradecyl)­phosphonium tris(pentafluoroethyl)­trifluorophosphate	[C_32_H_68_P]^+^ [C_6_F_18_P]^–^	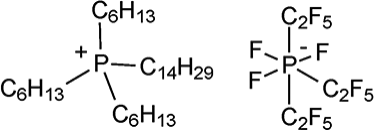
1-Butyl-1-methyl­pyrrolidinium bis(trifluoromethyl­sulfonyl)imide	[C_9_H_20_N]^+^ [C_2_F_6_NO_4_S_2_]^–^	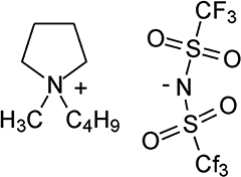
Perfluorononanoic acid	C_8_F_17_COOH	C_8_F_17_COO^–^
Polyethylene glycol diacid	HOOC-CH_2_O(CH_2_CH_2_O)_n_-CH_2_COOH	[HOOC-CH_2_O(CH_2_CH_2_O)*_n_*-CH_2_COO]^–^
Jeffamine D-400	H_2_N-(C_3_H_6_O)_n_CH_2_CH(CH_3_)­NH_2_	[H_2_N-(C_3_H_6_O)_n_CH_2_CH(CH_3_)-NH_3_]^+^
Jeffamine M-2005	CH_3_O-(C_2_H_4_O)_n_(C_3_H_6_O)_m_-NH_2_	[CH_3_O-(C_2_H_4_O)_n_(C_3_H_6_O)_m_-NH_3_]^+^

## Results and discussion

### Desorption of ionic liquid cations

Ionic liquids offer preformed ions in the liquid phase, and thus, present the perfect type of analytes to be used during the first steps of investigating ion desorption processes or ion source designs. The ionic liquids used here were already proven for this type of use by previous work.^14,50,58–61^ In particular the IL trihexyl(tetradecyl)phosphonium tris(pentafluoroethyl)trifluorophosphate, [C_32_H_68_P]^+^ [C_6_F_18_P]^–^, so far reliably yielded strong cation signals.

**Figure 2.**
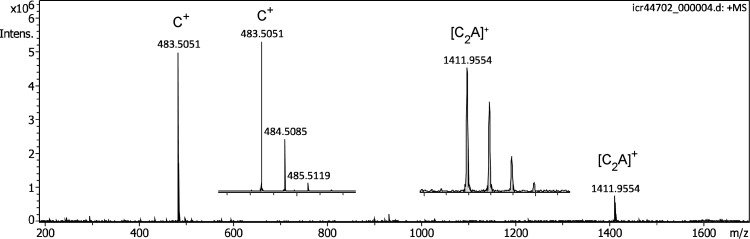
Positive-ion APFD spectrum of trihexyl(tetradecyl)phosphonium tris(pentafluoroethyl)trifluorophosphate, [C_32_H_68_P]^+^ [C_6_F_18_P]^–^ with the counter electrode at −5.0 kV showing peaks due to the intact cation, [C_32_H_68_P]^+^, *m/z* 483.5051, and the [C_2_A]^+^ cluster ion, [C_70_H_136_F_18_P_3_]^+^, *m/z* 1411.9954. Inserts provide the expanded views of the isotopic patterns of C^+^ and [C_2_A]^+^, respectively. The spectrum was obtained in configuration C1 and by accumulation of 16 transients of 512 k data points.

Here, scouting experiments were performed using the Bruker nanoESI source with its original flat polished stainless steel counter electrode. Provided the emitter was accurately aligned in front of the orifice, the IL delivered a very intensive signal of the cation, [C_32_H_68_P]^+^, *m/z* 483.5051 (calc. *m/z* 483.5053). Slight sideways shifts of the emitter would immediately result in a dramatic decrease or even loss of the signal. This strong dependance of mass spectral ion detection on the actual emitter position complicated the initial steps of this study. Nonetheless, once the position was optimized, the positive-ion APFD spectrum of the IL was encouraging. As the very first spectra were only acquired across the narrow *m/z* 150–1200 range, the formation of the [C_2_A]^+^ cluster ion, [C_70_H_136_F_18_P_3_]^+^, *m/z* 1411.9954 (calc. *m/z* 1411.9562), was only detected at a later stage of experiments covering up to *m/z* 2500 (Figure 2). The readout of the capillary current, normally an indicator of ion formation in ESI, also served as a rough measure of ion desorption from the emitter and typically was in the range of 50–150 µA, while discharges would occur when the value approached 1000 µA.

A counter electrode voltage of −5.0 to −5.6 kV yielded a signal for several minutes, while lower voltages caused the ion emission to drop. Re-adjusting the electrode voltage to higher values resulted in an immediate recovery of the emission (Electronic Supplement Figure S3). As the upper limit of the counter electrode potential also turned out to raise the risk of electric discharges between emitter and counter electrode, the voltage was normally set to +5.0 kV.

**Figure 3.**
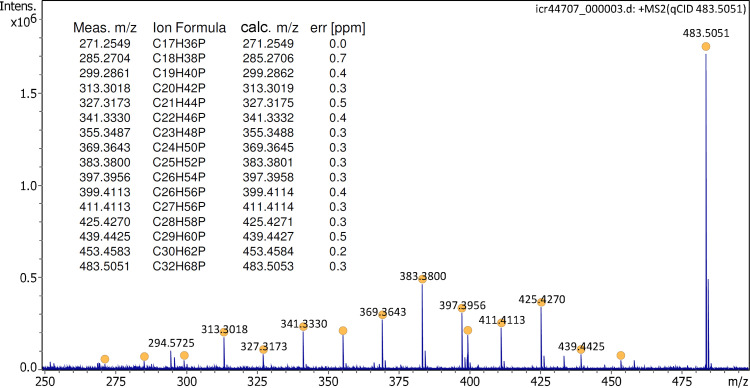
Tandem mass spectrum of the IL cation [C_32_H_68_P]^+^ at 50 V collision offset. Formulas are assigned to peaks marked with filled circles as listed in the insert. The spectrum was obtained in configuration C1 and by accumulation of 16 transients of 512 k data points.

Ion desorption from the activated emitter was stable enough to permit a tandem mass spectrum of the trihexyl(tetradecyl)phosphonium cation to be acquired. At a collision offset of 50 V, the CID spectrum of the IL cation exhibited numerous fragment ion peaks that could be assigned to alkane losses ranging from ethane loss, *m/z* 453.4583 (calc. 453.4584), to pentadecane loss, *m/z* 271.2549 (calc. 271.2549), and in addition, some hexene loss, *m/z* 399.4113 (calc. 399.4114) (Figure 3).

### Desorption of ionic liquid anions

**Figure 4.**
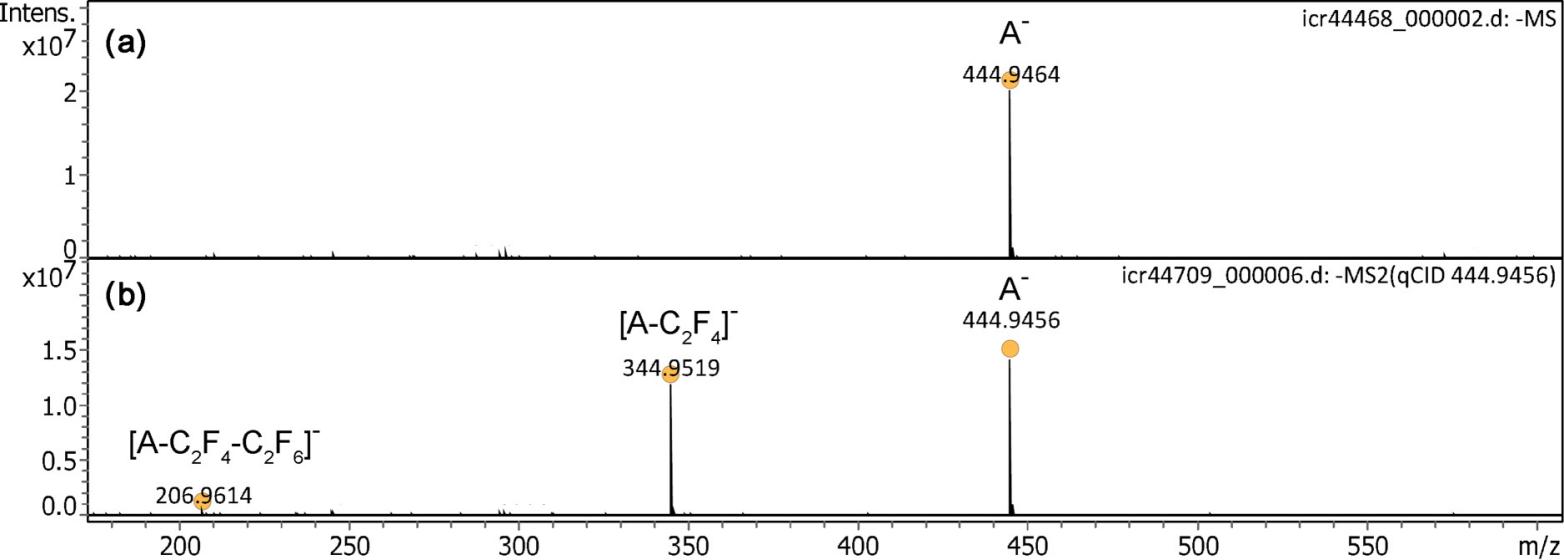
Negative-ion APFD spectra of trihexyl(tetradecyl)phosphonium tris(pentafluoroethyl)trifluorophosphate, [C_32_H_68_P]^+^ [C_6_F_18_P]^–^ with the counter electrode at +4.5 kV. (a) The spectrum shows the intact anion, [C_6_F_18_P]^–^, *m/z* 483.9464. (b) The tandem mass spectrum of the IL anion at 25 V collision offset reveals two fragment ions. The spectra were obtained in configuration C1 and by accumulation of 16 transients of 1 M data points.

Negative-ion field desorption had long been neglected and was only recently addressed again.^50,51^ When the polarity was switched to negative-ion mode, the IL anion, [C_6_F_18_P]^–^, *m/z* 444.9464 (calc. 444.9456) was observed at very high intensity (Figure 4). This is the first demonstration of negative-ion desorption from activated field emitters at atmospheric pressure. As in negative-ion mode discharges tended to occur at somewhat lower voltages than in positive-ion mode, a voltage of +4.5 to +5.0 kV was normally chosen. A comparison of anion peak intensities in spectra obtained at different ion source potentials ranging from +4.8 to +3.9 kV is shown in Figure S5. The actual onset of discharges depended on the exact distance between emitter and counter electrode and also on the shape of the latter (cf. next section). The distance was only judged from visual inspection via the monitor and typically in the order of 1–2 mm (Figure S2). The tandem mass spectrum of the anion at a collision offset of 25 V revealed two fragment ions at *m/z* 344.9519 and *m/z* 206.9614 that could be assigned to the formulas [C_4_F_14_P]^–^ (calc. 344.9520) by loss of C_2_F_4_ and [C_2_F_8_P]^–^ (calc. 206.9615) by further loss of C_2_F_6_ from this fragment, respectively.

### Ion source configurations

**Figure 5.**
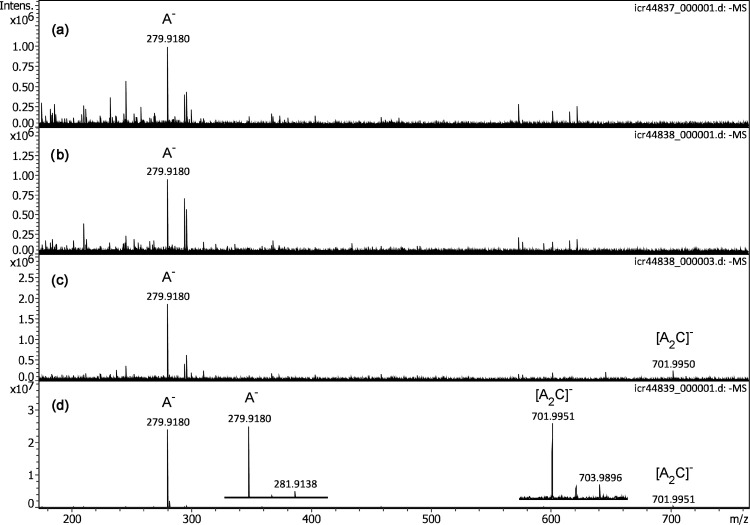
Negative-ion APFD spectra of 1-butyl-1-methylpyrrolidinium bis(trifluoromethyl­sulfonyl)imide, [C_9_H_20_N]^+^ [C_2_F_6_NO_4_S_2_]^–^, as obtained with configurations C2 to C4: (a) C2, spray shield +5.0 kV and cap +5.3 kV, (b) C3, only cap 5.3 kV, (c) C3, only cap +5.3 kV with drying gas, and (b) C4, bare transfer capillary +5.3 kV with drying gas. While the anion A^–^ is visible in spectra (a) to (d), the cluster ion [A_2_C]^–^ only appears when drying gas is on. Inserts in (d) show the expanded views of the isotopic patterns of A^–^ and [A_2_C]^–^, respectively. The spectra were obtained by accumulation of 16 transients of 512 k data points.

While it turned out that any API interface configuration was basically able to effect ion desorption from the emitter, the choice of the actual setup had dramatic influence on the final spectrum, most probably by variations in ion transmission rather than by notable changes in the ion currents from the emitter. This can be illustrated along the appearance of the negative-ion APFD spectrum of the ionic liquid 1-butyl-1-methylpyrrolidinium bis(trifluoromethylsulfonyl)imide, [C_9_H_20_N]^+^ [C_2_F_6_NO_4_S_2_]^–^ as obtained with configurations C2 to C4 (Figure 5). While noisy spectra were obtained with C2 and C3, some improvement could be achieved when a gentle flow of drying gas (1 l min^–1^ at 100 °C) was used. The simplest setup with the bare transfer capillary and drying gas at the same level delivered a tenfold increase in signal intensity. The IL anion A^–^, [C_2_F_6_NO_4_S_2_]^–^, *m/z* 279.9180 (calc. *m/z* 279.9178), was visible in all four spectra, whereas the cluster ion [A_2_C]^–^, [C_13_H_20_F_12_N_3_O_8_S_4_]^–^, *m/z* 701.9951 (calc. *m/z* 701.9947), only appeared when the drying gas flow was on. The expanded views of the isotopic patterns of A^–^ and [A_2_C]^–^ revealed the presence of sulfur due to the intensity of the respective [M + 2] peaks and their Δ(*m/z*) of 1.9958 (calc. ^32^S to ^34^S 1.9958 u) and Δ(*m/z*) of 1.9945, respectively. Among these configurations, the bare transfer capillary at +5.0 kV relative to the grounded emitter clearly delivered the best results and robust operation. It should also be noted that the negative-ion APFD spectra of these two ILs closely resembled those recently acquired in negative-ion vacuum LIFDI.^50^

### Perfluorononanoic acid and a matrix effect

**Figure 6.**
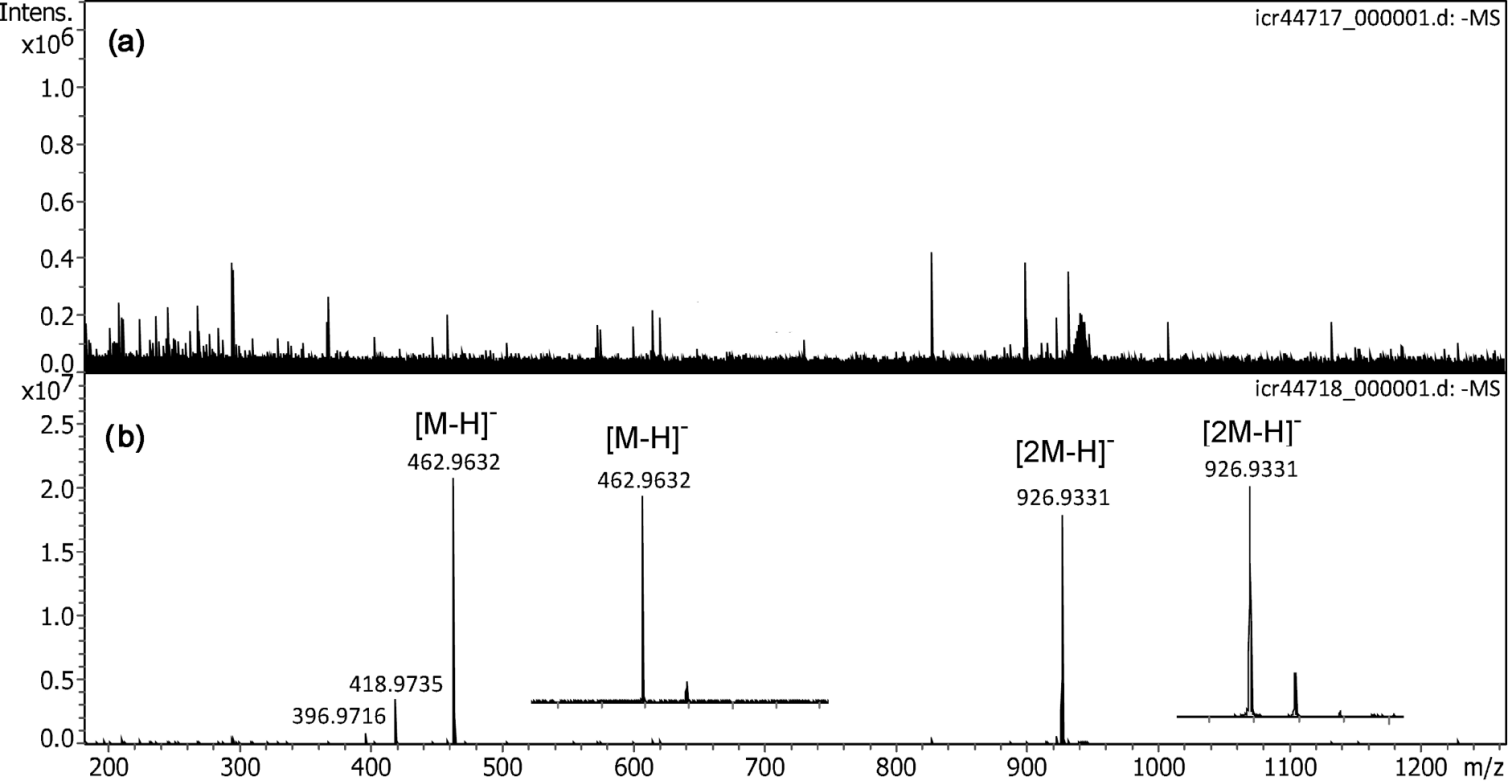
Negative-ion APFD spectra of perfluorononanoic acid (PFNA). (a) With solid PFNA on the emitter only baseline and some noise peaks did appear with the counter electrode at +5.0 kV. (b) When the same preparation was reused after addition of glycerol solution strong [M–H]^–^ and [2M–H]^–^ signals were observed even at an emitter voltage of just 4.0 kV. Inserts in (b) show the expanded views of the isotopic patterns of [M–H]^–^ and [2M–H]^–^, respectively. The spectra were obtained in configuration C3 by accumulation of 16 transients of 512 k data points.

In negative-ion DART, perfluoroalkanoic acids delivered intensive signals for both the deprotonated molecules, [M–H]^–^, and the [2M–H]^–^ cluster ions.^61,62^ PFNA was thus chosen to probe the ion desorption of solids. To do so, a solution of PFNA (2 mg ml^–1^ in MeOH) was applied to the emitter and allowed to crystallize. Up to a counter electrode voltage of +5.0 kV neither of the aforementioned ions could be observed (Figure 6). Early publications on negative-ion FD mentioned the use of medium polar oligomers as additives, i.e. as a matrix, to better achieve surface mobility of the analyte upon emitter heating while the matrix itself would not deliver negative ions.^63–67^ Recent work on negative-ion vacuum LIFDI also indicated a mobilization effect of waxy or viscous liquid matrices, e.g. the formation of I^–^ from iodine only in the presence of polyethylene glycol and the comparative ease of anion desorption from a commercial dishwashing detergent.^50^ Thus, a polar viscous liquid matrix was assumed to serve the purpose, in particular as this would not require emitter heating, which was not implemented in the present setup. Therefore, as in fast atom bombardment (FAB), glycerol was added to the sample preparation by adding 1 µl of glycerol solution (10 µl ml^–1^ in MeOH).^68,69^ The addition of the liquid matrix immediately allowed PFNA ion desorption to occur even at a lower voltage of just +4.0 kV. The spectrum showed intensive [M–H]^–^ and [2M–H]^–^ cluster ion signals at *m/z* 462.9632 (calc. for [C_9_F_17_O_2_]^–^ *m/z* 462.9632) and at *m/z* 926.9331 (calc. for [C_18_F_34_O_4_]^–^ *m/z* 926.9637), respectively (Figure 6). As in DART,^61^ two fragment ion peaks were observed at *m/z* 418.9735 and 396.9716, that could be assigned to [C_8_F_17_]^–^ (calc. 418.9734) and [C_8_F_15_O]^–^ (calc. 396.9715), respectively. The formula assignments were further substantiated by tandem MS of the [M–H]^–^ and the [2M–H]^–^ cluster ion (Figure S6). The tandem mass spectra were acquired in a single run without the need of supplying additional sample. This demonstrated that an intensive and long-lasting ion emission was achieved under these conditions. Most notably, the matrix effect could even be observed when glycerol was added to a preparation after a first trial with the neat PFNA.

Next, using configuration C2, the negative-ion APFD spectra of PFNA in the presence of glycerol were acquired to study the effect of desolvation gas flow and temperature on the relative abundance of these ions. It turned out that both more desolvation gas and higher temperature thereof translated into harsher conditions, i.e. in drastically reduced [2M–H]^–^ cluster ion abundance and in the more prominent appearance of the [C_8_F_17_]^–^ fragment ion formed by CO_2_ loss from the [M–H]^–^ ion (Figure S7). Furthermore, higher temperature of the desolvation gas caused an overall loss in signal intensity, e.g. the most intensive signal within a spectrum dropped by three orders of magnitude when going from 1.0 l min^–1^ at 100 °C to 2.0 l min^–1^ at 200 °C, whereas a mere increase of desolvation gas flow from 1.0 l min^–1^ at 100 °C to 2.0 l min^–1^ at 100 °C only led to a loss by a factor of ten. Overall, using 1.0–1.5 l min^–1^ at 100 °C appeared as the optimum setting in combination with C2.

### Application to oligomers

To further explore the range of potential applications of APFD and to test the relevance of using a matrix to support ion desorption some oligomers were examined.

**Figure 7.**
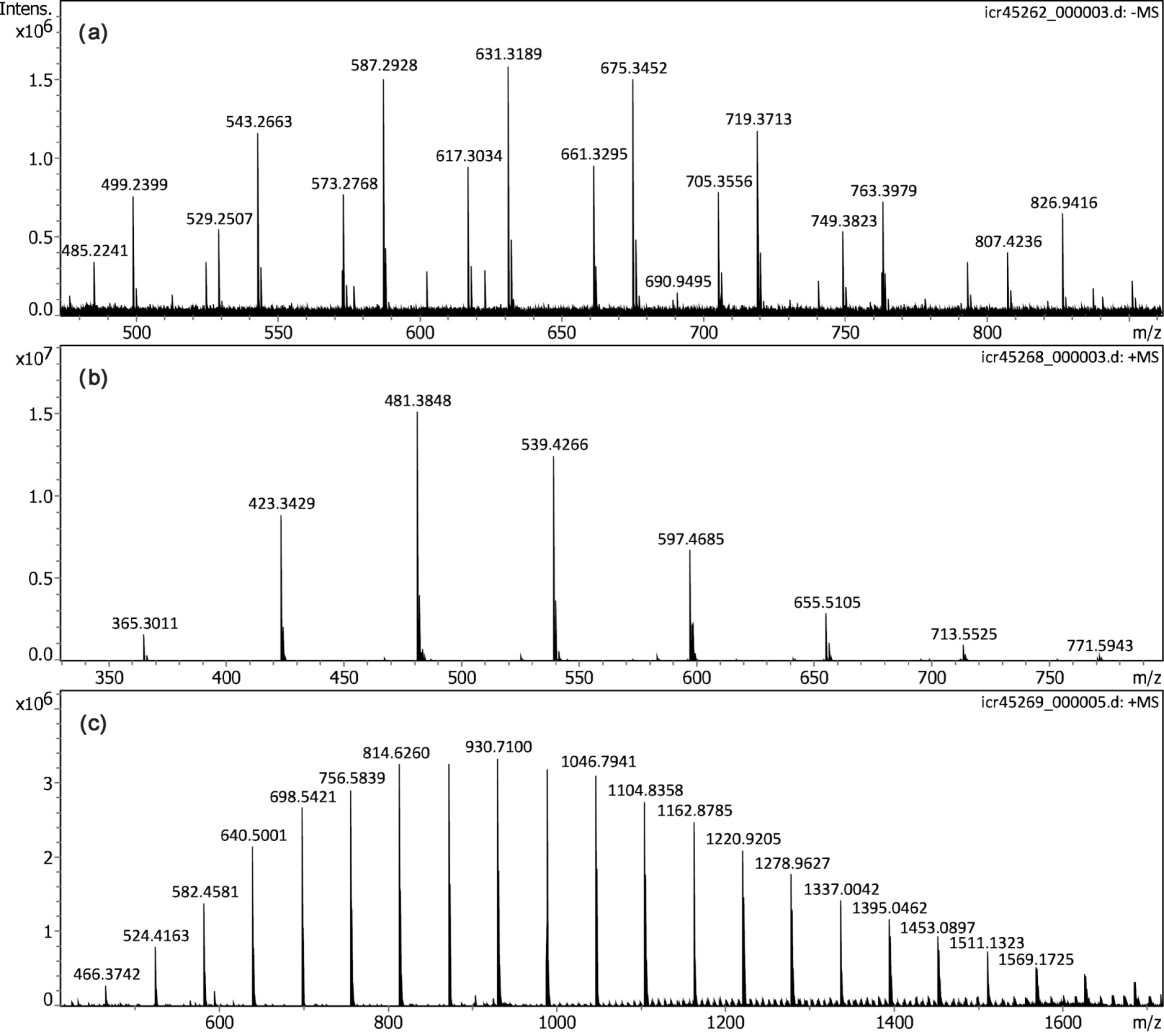
APFD spectra of oligomers: (a) Negative-ion spectrum of polyethylene glycol diacid (PEGDA-600) forming ions by deprotonation (glycerol matrix, C4, +5.3 kV, dry gas at 1.0 l min^–1^ and 100 °C). (b) Positive-ion spectrum of neat Jeffamine D-400 (C2, shield −4.5 kV, cap −5.0 kV, dry gas 1.2 l min^–1^ and 100 °C) showing protonated molecules. (c) Positive-ion spectrum of neat Jeffamine M-2005 (C2, shield −4.5 kV, cap −5.0 kV, dry gas 1.2 l min^–1^ and 100 °C) also showing a series of protonated molecules.

Polyethylene glycol diacid (PEGDA-600) was expected to form [HOOC-CH_2_O(CH_2_CH_2_O)*_n_*-CH_2_COO]^–^ ions by deprotonation, and was thus analyzed by negative-ion APFD. While being a highly viscous liquid at room temperature, PEGDA-600 still required the addition of glycerol to show reasonable level of ion desorption. The particular sample of PEGDA-600 was known from DART work.^53^ Here, it showed the above ion series plus a second set of ions having one methylene unit in addition, e.g. probably [CH_3_OOC-CH_2_O(CH_2_CH_2_O)*_n_*-CH_2_CH_2_COO]^–^ or [HOOC-CH_2_O(CH_2_CH_2_O)*_n_*-CH_2_CH_2_COO]^–^ ions (Figure 7). This second series appeared at higher intensity than the first. Nonetheless, both series were also present in the negative-ion DART spectrum even though the first was dominant there.^53^ The APFD spectrum with formula assignments is provided in Figure S8 and the negative-ion ESI spectrum for comparison in Figure S9, the latter being extremely close in appearance to the APFD spectrum.

Some basic poly(propylene glycols), commercially available under the trade name Jeffamine, were previously studied as internal mass calibrants in MALDI-TOF-MS.^70^ According to their basic endgroups, these Jeffamines delivered high yields of [M + H]^+^ ions. Here, Jeffamines D-400 and M-2005, both viscous liquids at room temperature, were subjected to positive-ion APFD analysis. Using setup C2 (shield at −4.5 kV, cap at −5.0 kV, dry gas at 1.2 l min^–1^ and 100 °C) and without the addition of glycerol, both delivered intensive spectra revealing the respective series of protonated molecules. The ion series were evenly spaced at Δ(*m/z*) = 58.0419 as expected for the C_3_H_6_O monomer unit (Figure 7). The additional ions appearing in case of Jeffamine M-2005 from > *m/z* 1000 between the major ion series at tighter spacings may be due to various cluster ion composition and were not further investigated at this stage of the work. It should be noted that the *m/z* range beyond 1800 covered in the APFD spectrum of Jeffamine M-2005 notably exceeded that previously observed in the MALDI spectra MS.^70^ The APFD spectra and for comparison also the positive-ion ESI spectra of both Jeffamines in methanol, each with formula assignments, are provided separately (Figures S10 to S13). As with the PEGDA before, APFD and ESI spectra exhibited very close resemblance.

### Thoughts on the mechanism of ion desorption

Concerning the mechanism of ion desorption, only some thoughts can be presented at this point. So far, even-electron ions were exclusively observed here while FI yielding molecular ions did not occur or at least the products of it were not detected. As is the case in ESI, ion desorption from the field emitters was exclusively observed when preformed ions where present.

The release of ions from the activated field emitters clearly required strong electric fields as ion desorption only occurred at kilovolt potentials between emitter and counter electrode. Like in ESI, this process delivered either positive or negative ions, depending on the polarity of the potential and on the properties of the analyte. Ions were solely desorbed from the (viscous) liquid phase, which points towards ion desorption as described by the processes of ion evaporation or ion desolvation, i.e. the role of the electric field is only to effect desorption of preformed ions from the surface into the gas phase. The field strength required for desorption of ions from the condensed phase is far below that for FI.^7,8,71–73^

In (vacuum) FD-MS, field-induced desolvation^74–76^ and ion evaporation^77,78^ were suggested to explain ion desorption. Both models assume that ions are already present in the condensed phase and are subsequently desorbed into the gas phase by action of the electric field. The model of field-induced desolvation was based in the observation of microscopic protuberances from sample layers.^74,75^ On the other hand, the release of ions via spray generation from microscopic Taylor cones as in nanoESI or in case of ESI performed from liquid films covering the surface of sharp needles and similar tips can be considered.^44–47^ The viscous matrix used here delivered the mobility for those processes.

The processes reported here, i.e. the release of (preformed) ions from the condensed phase, probably via microscopic protuberances, point toward some similarity to Taylor cone formation in ESI. It now appears that there is something like a continuum from “pure FD” to “pure ESI”. In other words, ion formation in what is termed here as atmospheric pressure field desorption proceeds in the transition zone from nanoESI from a surface to ion evaporation from field emitters.

## Conclusion

Atmospheric pressure field desorption (APFD) has been realized by adaptation of a nanoESI source to serve for precise emitter positioning in front of the atmospheric pressure interface of an FT-ICR mass spectrometer. The setup enabled ion desorption from 13-µm activated tungsten emitters at atmospheric pressure and essentially under ambient conditions. Both positive-ion, and for the first time, negative-ion desorption was observed. APFD of ionic and highly polar analytes turned out to be robust and reproducible. Four different configurations of the atmospheric pressure interface were explored and either of these turned out to serve the purpose, even though the simplest setup (C2) worked best.

It appeared as a necessity for APFD that preformed ions are present in a viscous liquid that is spread across the large surface of the activated field emitter, i.e. some surface mobility on the emitter is prerequisite for ion desorption to occur. In case of solid analytes, the use of a liquid matrix like glycerol provided the surface mobility needed. Once ion desorption occurred, it was generally intensive in either positive-ion or negative-ion modes. The compounds examined have all been characterized by accurate mass, and in part, by tandem MS.

Admittedly, the technique described here may not be able to compete with other established atmospheric pressure ionization techniques. Nonetheless, the present study fills a gap in fundamentals of ion desorption processes that is roughly located in the center of a triangle spanned by field desorption, electrospray ionization, and ambient MS. A continuation of this work could explore other matrices that might enable a wider range of samples to be analyzed. So far, singly charged even-electron ions were exclusively observed here. However, others have shown that molecular ion formation is not impossible.^6^ Further, life-time requirements and softness of ion transfer do play a crucial role when it comes to the transmission of intact molecular ions.^15^ Thus, one could expect to find suitable conditions in the future. Technical improvements might address the ease of emitter positioning and eventually the implementation of an emitter heating current.

## Supplemental Material

sj-pdf-1-ems-10.1177_14690667221133388 - Supplemental material for Desorption of positive and negative ions from activated field emitters at atmospheric pressure Click here for additional data file. Supplemental material, sj-pdf-1-ems-10.1177_14690667221133388 for Desorption of positive and negative ions from activated field emitters at atmospheric pressure by Jürgen H. Gross in European Journal of Mass Spectrometry
