# Supplementary material for: Desorption of positive and negative ions from activated field emitters at atmospheric pressure
Source: Eur J Mass Spectrom (Chichester). 2022 Oct 18;29(1):21–32. doi: 10.1177/14690667221133388 (PMC9903004; doi:10.1177/14690667221133388)
Supplement: sj-pdf-1-ems-10.1177_14690667221133388 - Supplemental material for Desorption of positive and negative ions from activated field emitters at atmospheric pressure [file sj-pdf-1-ems-10.1177_14690667221133388.pdf]

Desorption of positive and negative ions from activated field emitters at atmospheric pressure

Supplementary Data

Jürgen H. Gross

ORCID 0000-0003-0748-2535

Address

Dr. Jürgen H. Gross

Institute of Organic Chemistry

Heidelberg University

Im Neuenheimer Feld 270

69120 Heidelberg

Germany

email: juergen.gross@oci.uni-heidelberg.de

phone: +49/6221/54-8409

fax: +49/6221/54-4205

Fig. S1. a) Setup for atmospheric pressure field desorption (APFD) based on the Bruker nanoESI source. **b)** Custom-built emitter holder. **c)** After releasing the small clip, the black part of the source can be opened as in **d)** via a hinge to access the emitter. Also cf. effects of switching on and off via opening and closing in Fig. S4.

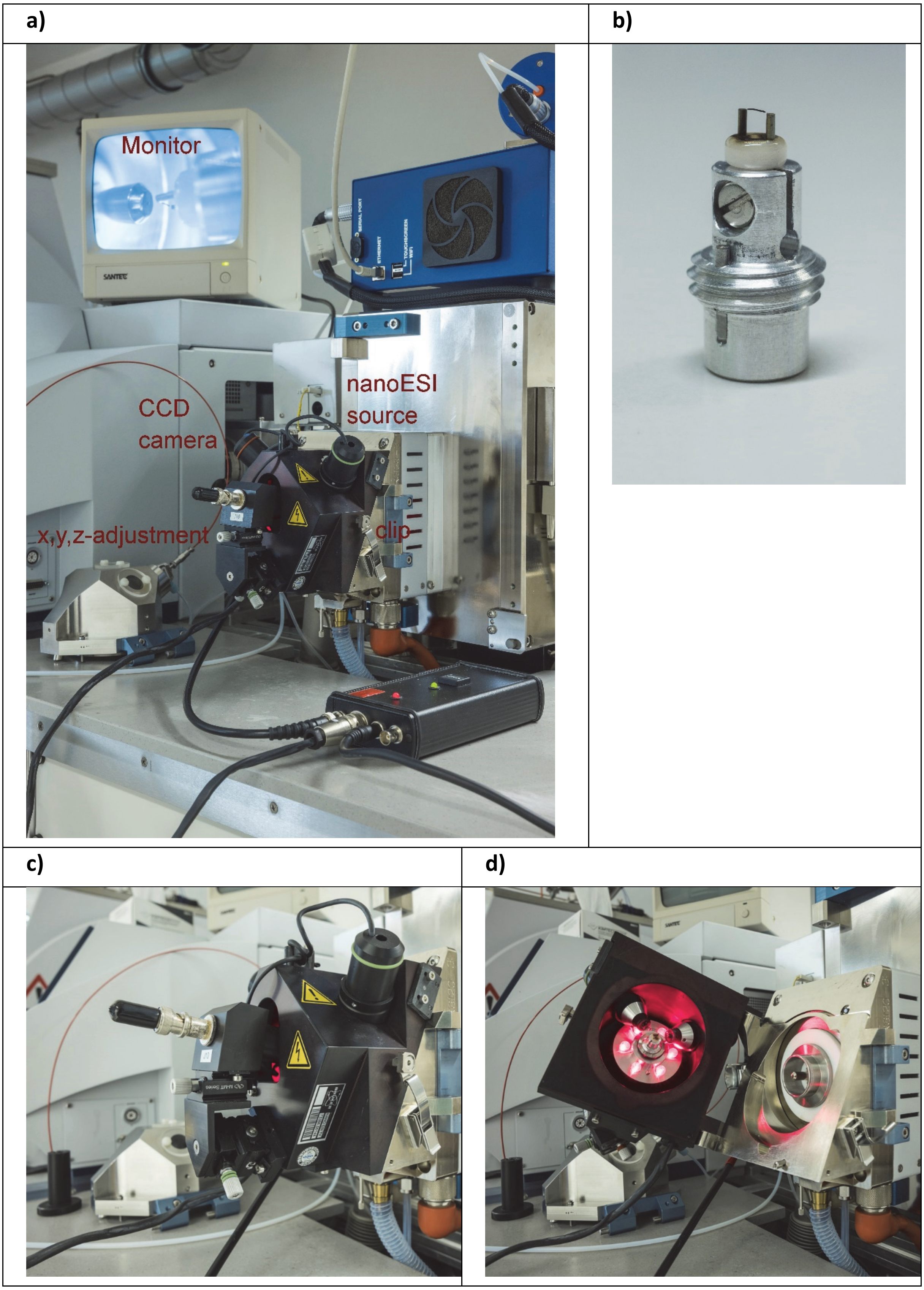

Fig. S2. Photographs of the monitor display delivered by the built-in CCD camera of the nanoESI source. The configurations of the activated emitter in front of the counter electrode provided by the Bruker API interface in comparison: **a)** dedicated flat and polished spray shield of the nanoESI source (C1), **b)** conventional ESI source entrance with complete spray shield and metal cap on the transfer capillary behind it (C2), **c)** rounded cap only on the transfer capillary (C3), and **d)** bare transfer capillary (C4). While either of configurations C1 to C4 allowed ion desorption to occur, C2 turned out to be the least critical in terms of emitter position and providing the best ion transmission, too. Thus, C2 was mainly used during later stages of this work. C4 also worked reasonably well.

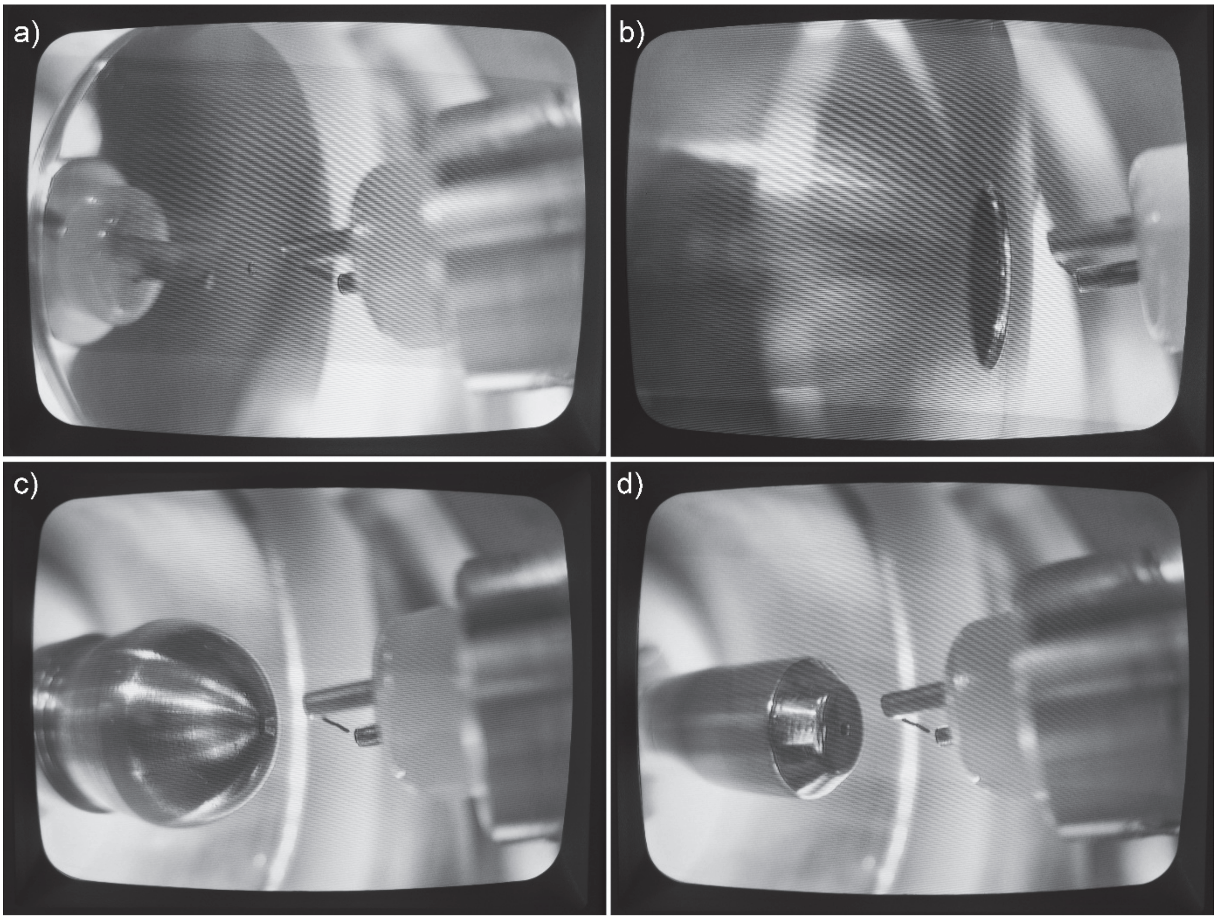

Fig. S3. Positive-ion atmospheric pressure field desorption (APFD) of the ionic liquid tri-hexyl(tetradecyl)phosphonium tris(pentafluoroethyl)trifluorophosphate, $[C_{32}H_{68}P]^+$ $[C_6F_{18}P]^-$, at different counter electrode voltages. All spectra shown were obtained in one series from a single application of sample (2 µl at 1 µl ml^{-1} in MeOH) to the emitter. All alignments were thus fixed during the run. The cation signal at *m/z* 483.5039 exhibited strong dependence on the actual electrode voltage and recovered as soon as the value was increased again. The data was obtained using the Bruker nanoESI flat stainless steel counter electrode (C1).

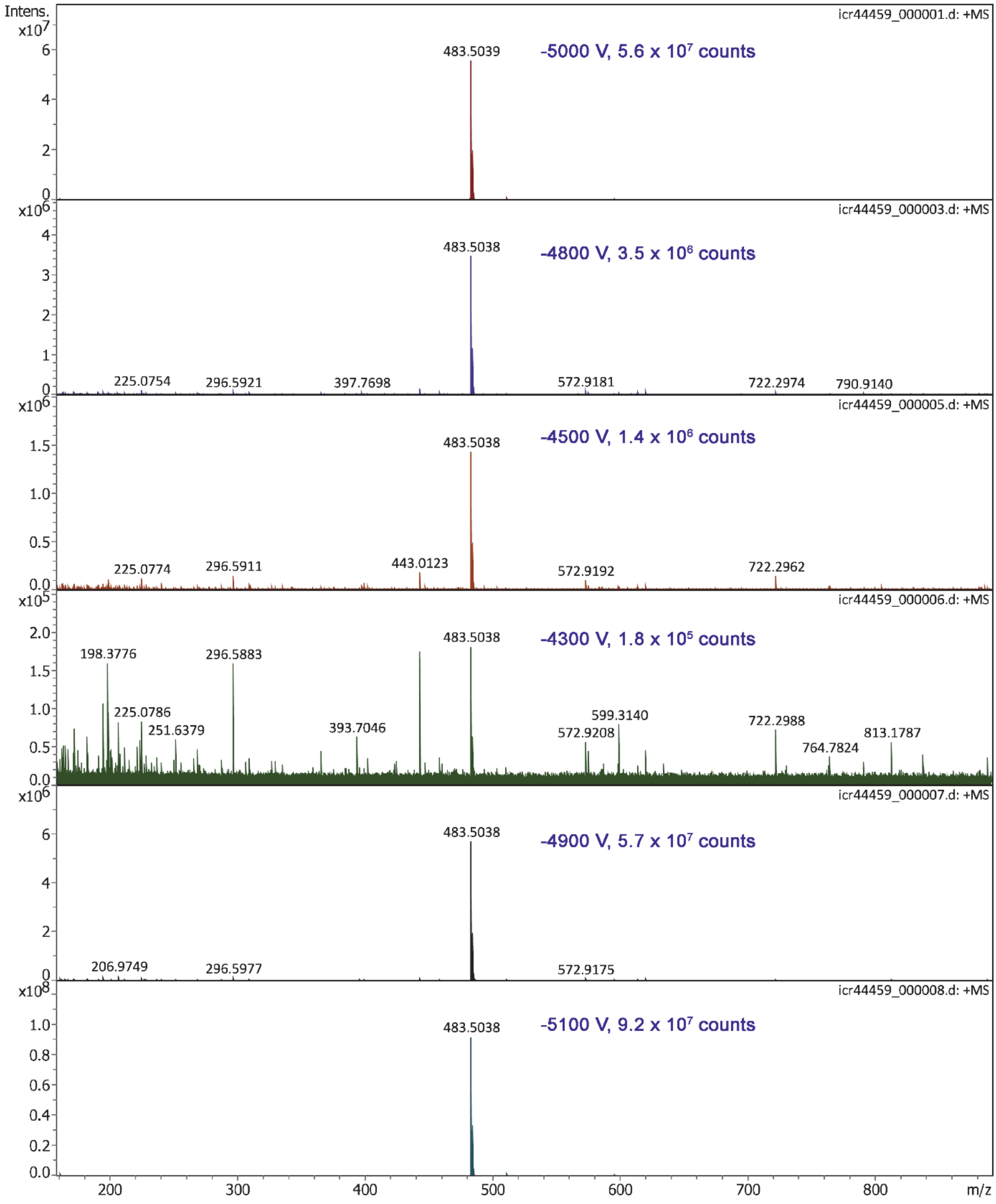

Fig. S4. Positive-ion atmospheric pressure field desorption (APFD) of the ionic liquid tri-hexyl(tetradecyl)phosphonium tris(pentafluoroethyl)trifluorophosphate, $[C_{32}H_{68}P]^+$ $[C_6F_{18}P]^-$, while the door of the Bruker nanoESI source was closed and opened in alteration (cf. Fig. S1 c) and d). Positioning of the emitter was highly reproducible and emission immediately started as soon as the emitter was repositioned in front of the orifice. The high voltage of –5.0 kV was on at all times during this run. This also demonstrated robustness of the method once the emitter was correctly aligned. Both C^+ and $[C_2A]^+$ peaks of the IL were observed.

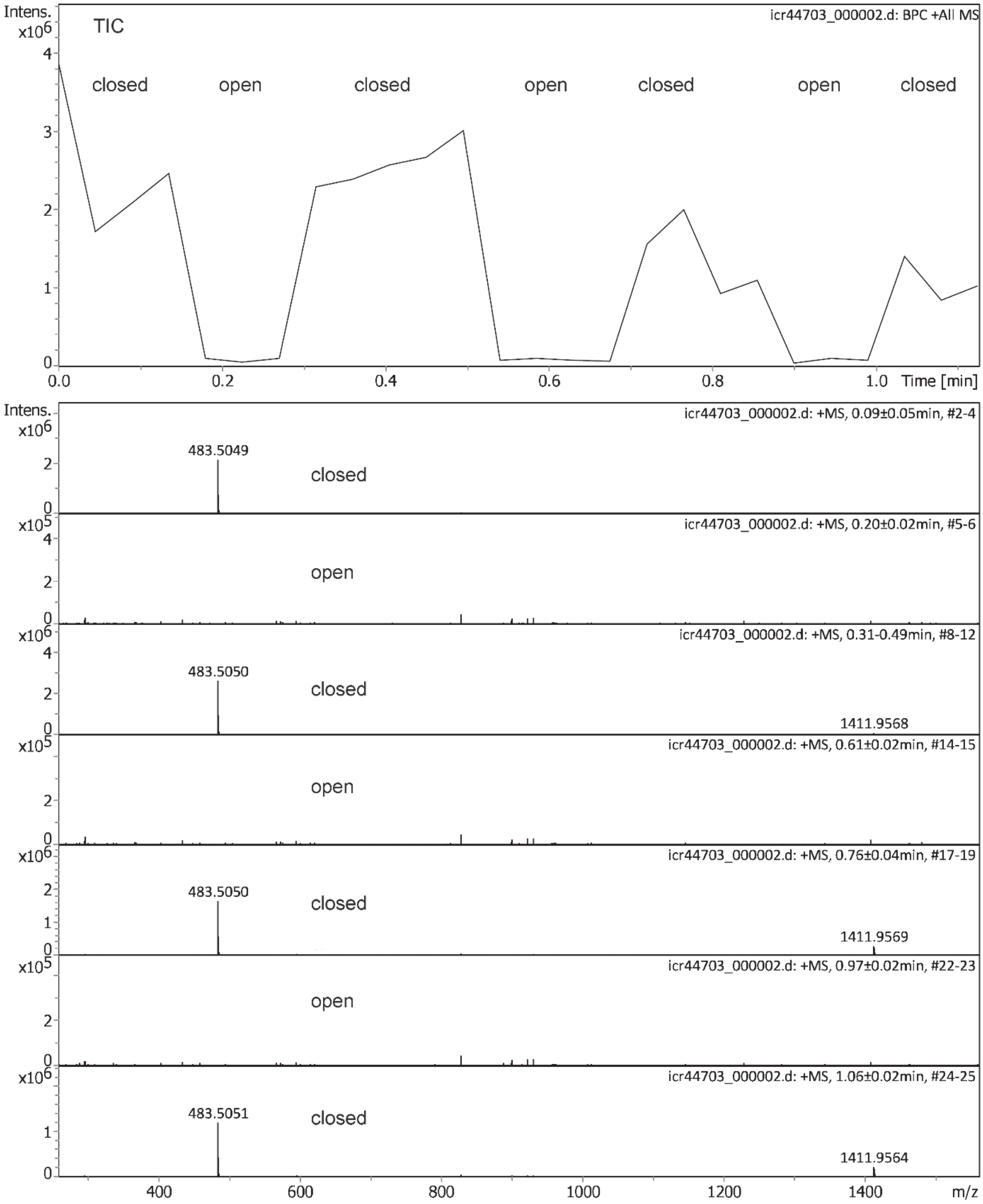

Fig. S5. Negative-ion APFD spectra of trihexyl(tetradecyl)phosphonium tris(pentafluoroethyl)trifluorophosphate, $[C_{32}H_{68}P]^{+}$ $[C_6F_{18}P]^{-}$ with the counter electrode at various potentials. The anion peak intensity decreases at lower values. To avoid discharges, a setting of around +4.5 kV was used in practice. The inserts show the expanded isotopic patterns of the anion peak. Spectra were obtained by application of 2 µl IL solution at 1 µl ml^{-1} in MeOH to the emitter.

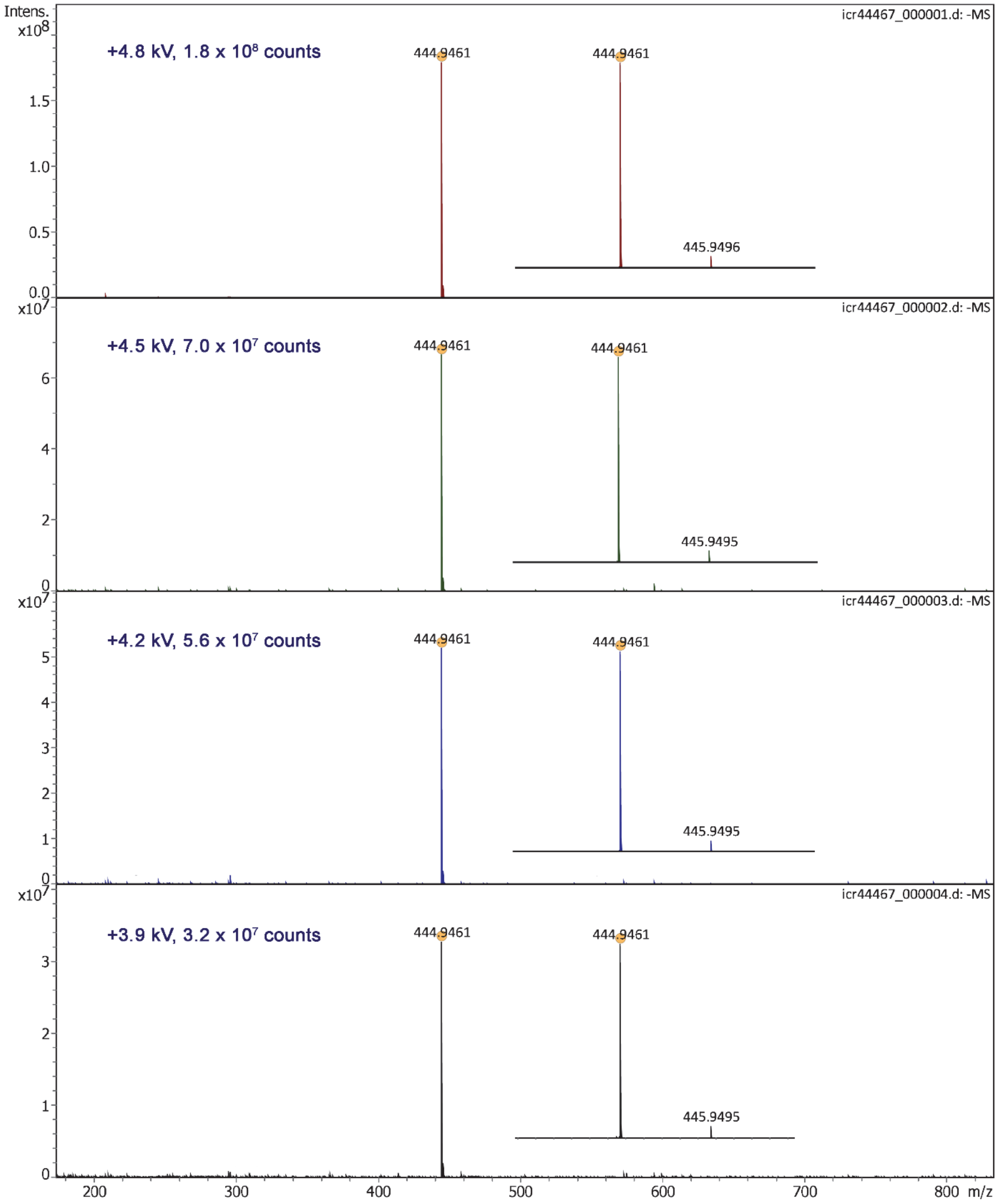

Fig. S6. Negative-ion APFD tandem mass spectra of perfluorononanoic acid as acquired at +4.0 kV where 1 µl of PFNA solution (2 mg ml^{-1} in MeOH) was applied to the emitter together with 1 µl of glycerol solution (10 µl ml^{-1} in MeOH). The tandem mass spectra show (*top*) isolation and some metastable ion fragmentation of the $[2M–H]^{-}$ cluster ion, (*middle*) CID of this ion at 5 V collision offset, and (*bottom*) CID of the $[M–H]^{-}$ ion at 5 V collision offset. Formula assignments are included as inserts in the spectrum plots. The spectra were obtained in configuration C3 by accumulation of 16 transients of 512 k data points.

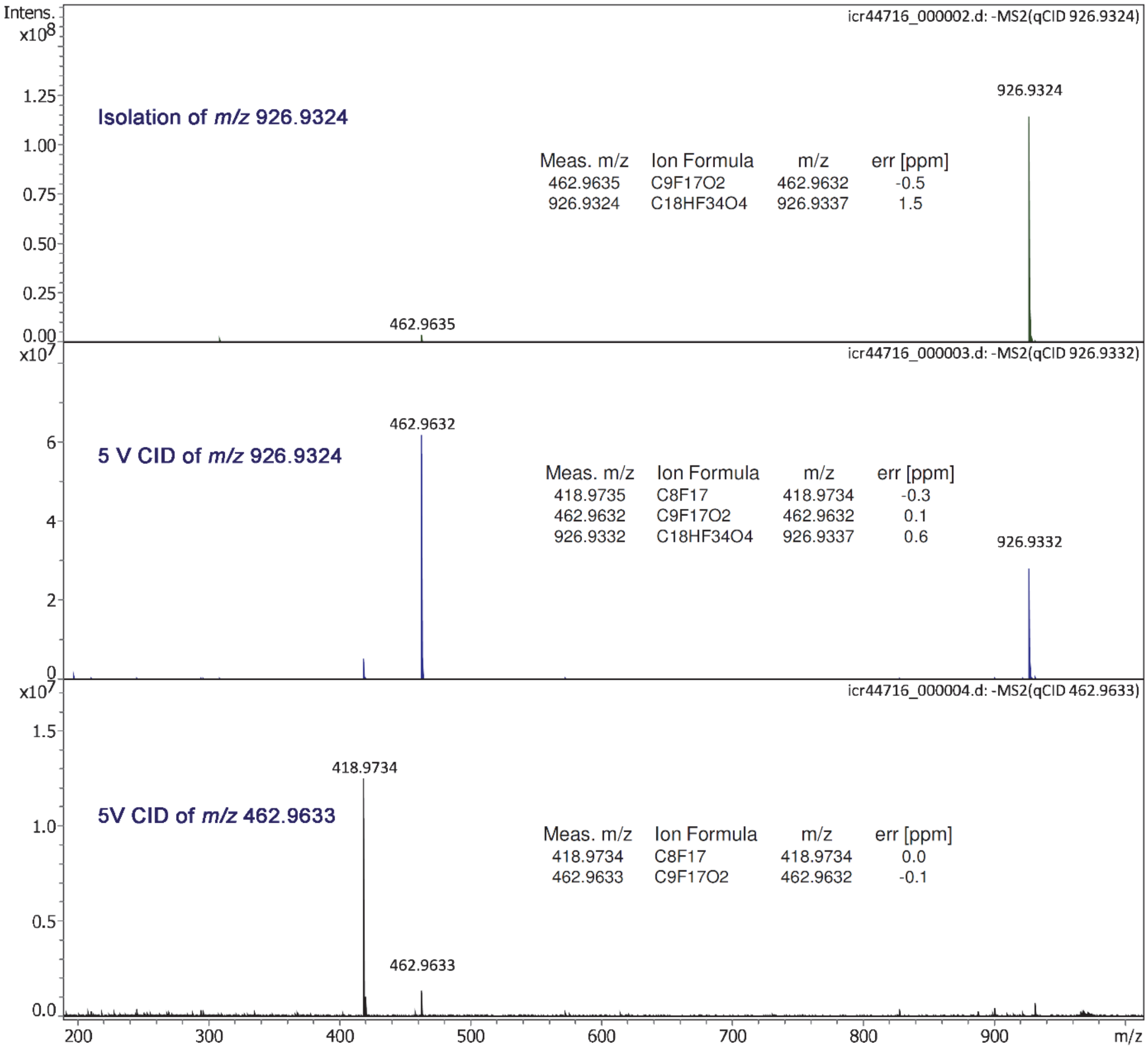

Fig. S7. Negative-ion APFD spectra of PFNA acquired using C2 (shield +3.8 kV, cap +4.2 kV) with 1 µl of PFNA solution (2 mg ml^{-1} in MeOH) plus 1 µl of glycerol solution (10 µl ml^{-1} in MeOH) applied to the emitter. Desolvation gas flows and temperatures are annotated to the spectra. More desolvation gas and higher temperature result in harsher conditions, i.e., reduced [2M–H]$^{-}$ cluster ion abundance and in more prominent appearance of the [C_8F_{17}]$^{-}$ fragment ion formed by CO_2 loss from the [M–H]$^{-}$ ion. **a)** The upper three spectra combine rising temperature and gas flow while **b)** the lower set shows the effect of increased flow alone. Hot gas diminishes the cluster abundance most but also goes along with much reduced intensity.

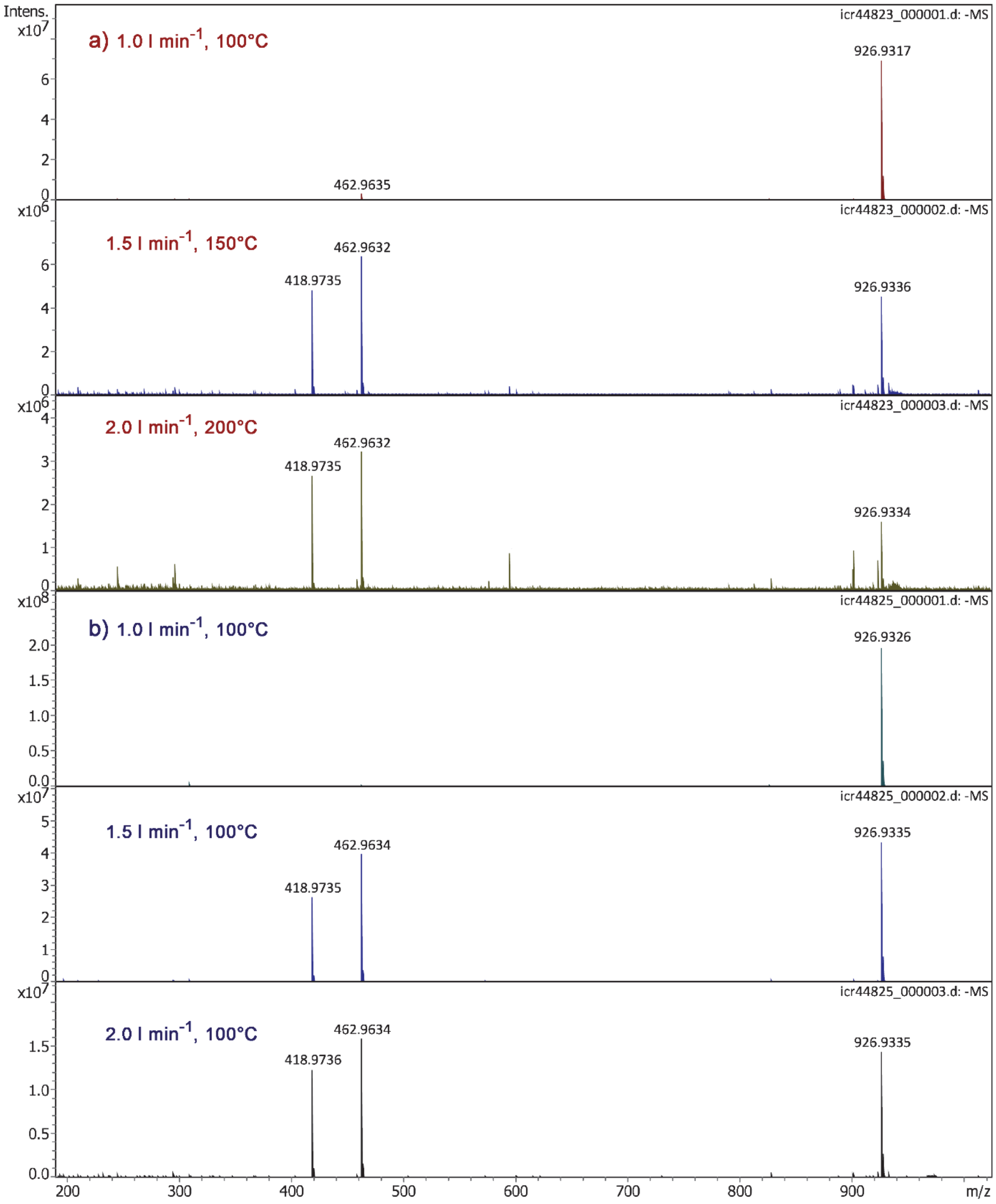

Fig. S8. Negative-ion APFD spectrum of polyethylene glycol diacid (PEGDA-600) in the presence of glycerol matrix. The spectrum was acquired using C4, a voltage of +5.3 kV, and dry gas at 1.0 l min^{-1} and 100 °C.

Two ion series are observed, the expected series of [HOOC-$CH_2O(CH_2CH_2O)_n$-CH_2COO]$^-$ ions plus a series of ions having one methylene unit in addition, e.g., [CH_3OOC-$CH_2O(CH_2CH_2O)_n$-CH_2CH_2COO]$^-$ or eventually [HOOC-$CH_2O(CH_2CH_2O)_n$-CH_2CH_2COO]$^-$ ions. The $\Delta(m/z)$ values are annotated to several peak pairs and clearly indicate C_2H_4O (calc. 44.0262) and CH_2 (calc. 14.0156), respectively. Formula assignments of the ion series are also provided.

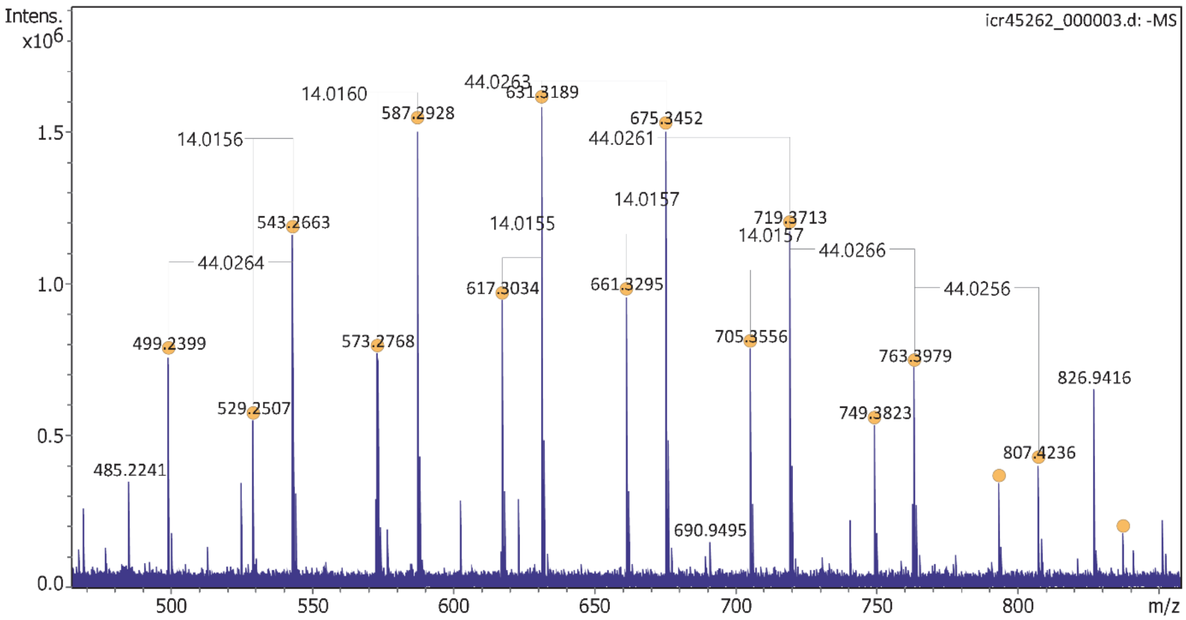

Meas. m/z	Ion Formula	m/z	err [ppm]	mSigma	rdb	e⁻ Conf	N-Rule
485.2241	C20H37O13	485.2240	-0.3	n.a.	2.0	even	ok
499.2399	C21H39O13	499.2396	-0.6	30.9	2.0	even	ok
529.2507	C22H41O14	529.2502	-0.9	n.a.	2.0	even	ok
543.2663	C23H43O14	543.2658	-0.8	31.1	2.0	even	ok
573.2768	C24H45O15	573.2764	-0.7	34.1	2.0	even	ok
587.2928	C25H47O15	587.2920	-1.2	35.1	2.0	even	ok
617.3034	C26H49O16	617.3026	-1.3	43.7	2.0	even	ok
631.3189	C27H51O16	631.3183	-1.1	7.9	2.0	even	ok
661.3295	C28H53O17	661.3288	-1.1	43.0	2.0	even	ok
675.3452	C29H55O17	675.3445	-1.1	8.4	2.0	even	ok
705.3556	C30H57O18	705.3550	-0.7	47.7	2.0	even	ok
719.3713	C31H59O18	719.3707	-0.9	49.1	2.0	even	ok
749.3823	C32H61O19	749.3813	-1.4	53.1	2.0	even	ok
763.3979	C33H63O19	763.3969	-1.3	54.4	2.0	even	ok
793.4087	C34H65O20	793.4075	-1.6	57.7	2.0	even	ok
807.4236	C35H67O20	807.4231	-0.6	59.7	2.0	even	ok
837.4351	C36H69O21	837.4337	-1.7	n.a.	2.0	even	ok

Fig. S9. Negative-ion ESI spectrum of polyethylene glycol diacid (PEGDA-600) in methanol. The spectrum was acquired using the above solution diluted 1:100 in methanol which was infused from a syringe at 8 µl min^{-1}.

Both relative intensity of these two ion series and the *m/z* range covered closely correspond to the APFD spectrum. Formula assignments of both ion series are also provided. The spectrum nicely corresponds to the APFD results.

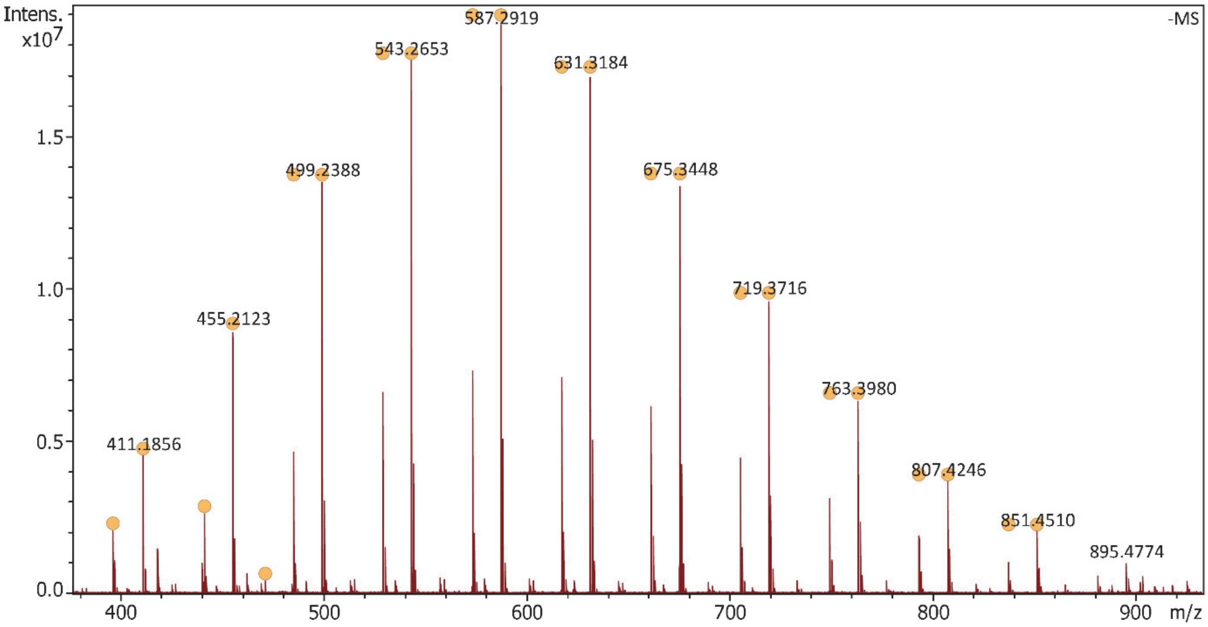

Meas. m/z	Ion Formula	m/z	err [ppm]	mSigma	rdb	e⁻ Conf	N-Rule
396.1984	C17H32O10	396.2001	4.2	70.0	1.5	odd	ok
411.1856	C17H31O11	411.1872	3.8	11.1	2.0	even	ok
441.1966	C18H33O12	441.1978	2.6	14.0	2.5	even	ok
455.2123	C19H35O12	455.2134	2.4	8.9	2.5	even	ok
471.2440	C20H39O12	471.2447	1.5	n.a.	1.5	even	ok
485.2232	C20H37O13	485.2240	1.7	10.6	2.0	even	ok
499.2388	C21H39O13	499.2396	1.7	12.7	2.5	even	ok
529.2497	C22H41O14	529.2502	0.8	14.2	2.0	even	ok
543.2653	C23H43O14	543.2658	1.0	10.1	2.5	even	ok
573.2764	C24H45O15	573.2764	-0.1	7.6	2.0	even	ok
587.2919	C25H47O15	587.2920	0.3	8.9	2.5	even	ok
617.3029	C26H49O16	617.3026	-0.5	7.3	2.0	even	ok
631.3184	C27H51O16	631.3183	-0.2	8.3	2.5	even	ok
661.3295	C28H53O17	661.3288	-1.0	8.1	2.0	even	ok
675.3448	C29H55O17	675.3445	-0.5	8.2	2.5	even	ok
705.3560	C30H57O18	705.3550	-1.3	2.1	2.0	even	ok
719.3716	C31H59O18	719.3707	-1.2	8.6	2.5	even	ok
749.3823	C32H61O19	749.3813	-1.5	6.1	2.0	even	ok
763.3980	C33H63O19	763.3969	-1.4	4.6	2.5	even	ok
793.4090	C34H65O20	793.4075	-2.0	18.1	2.5	even	ok
807.4246	C35H67O20	807.4231	-1.8	8.1	2.5	even	ok
837.4352	C36H69O21	837.4337	-1.8	16.3	2.5	even	ok
851.4510	C37H71O21	851.4493	-2.0	16.0	2.5	even	ok

Fig. S10. Positive-ion APFD spectrum of the basic poly(propylene glycol) Jeffamine D-400. Jeffamine D-400, a viscous liquid at room temperature, was subjected to APFD analysis using setup C2 (shield at –4.5 kV, cap at –5.0 kV, dry gas at 1.2 l min^{-1} and 100 °C) and without the addition of glycerol matrix. The neat sample delivered an intensive spectrum revealing the series of protonated molecules spaced at $\Delta(m/z)$ = 58.0419 due to the C_3H_6O monomer unit. Formula assignments of the ion series are also provided.

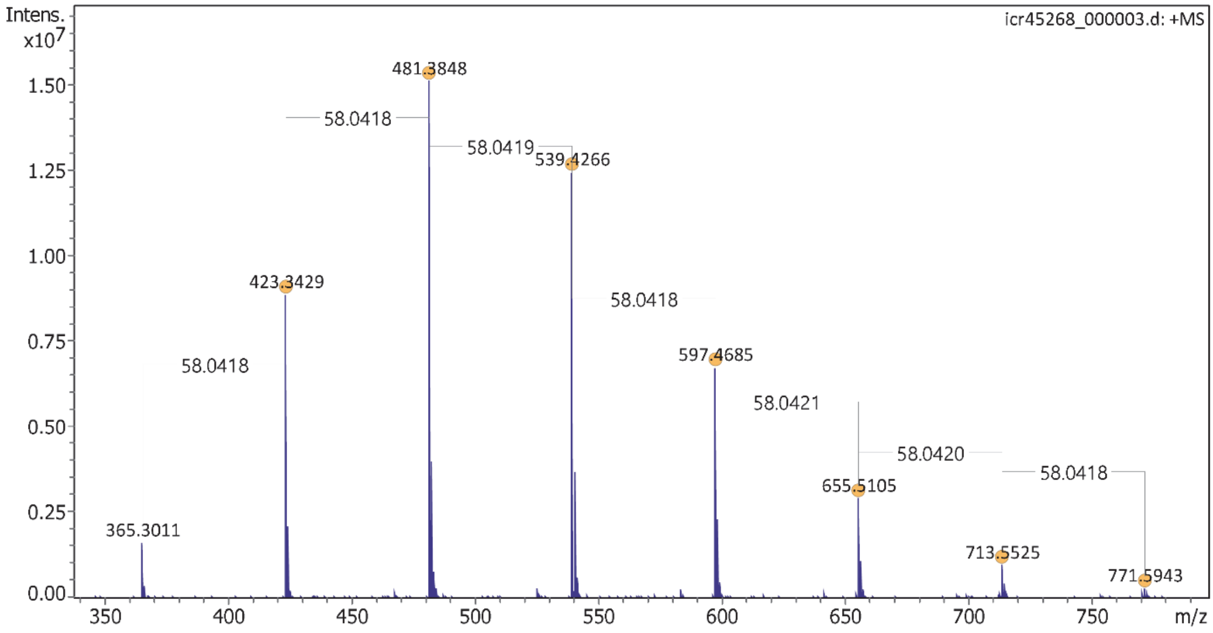

Meas. m/z	Ion Formula	m/z	err [ppm]	mSigma	rdb	e⁻ Conf	N-Rule
365.3011	C18H41N2O5	365.3010	-0.3	8.2	-0.5	even	ok
423.3429	C21H47N2O6	423.3429	-0.1	10.6	-0.5	even	ok
481.3848	C24H53N2O7	481.3847	-0.1	12.4	-0.5	even	ok
539.4266	C27H59N2O8	539.4266	-0.1	9.3	-0.5	even	ok
597.4685	C30H65N2O9	597.4685	-0.0	5.9	-0.5	even	ok
655.5105	C33H71N2O10	655.5103	-0.3	6.6	-0.5	even	ok
713.5525	C36H77N2O11	713.5522	-0.5	10.8	-0.5	even	ok
771.5943	C39H83N2O12	771.5941	-0.4	14.0	0.0	even	ok

Fig. S11. Positive-ion ESI spectrum of the basic poly(propylene glycol) Jeffamine D-400 where the above solution has been diluted 1:100 in methanol which was infused from a syringe at 8 µl min^{-1}. The relative intensity of the peaks along the ion series and the *m/z* range covered closely correspond to the APFD spectrum. Formula assignments of the ion series are also provided and also correspond to the APFD results.

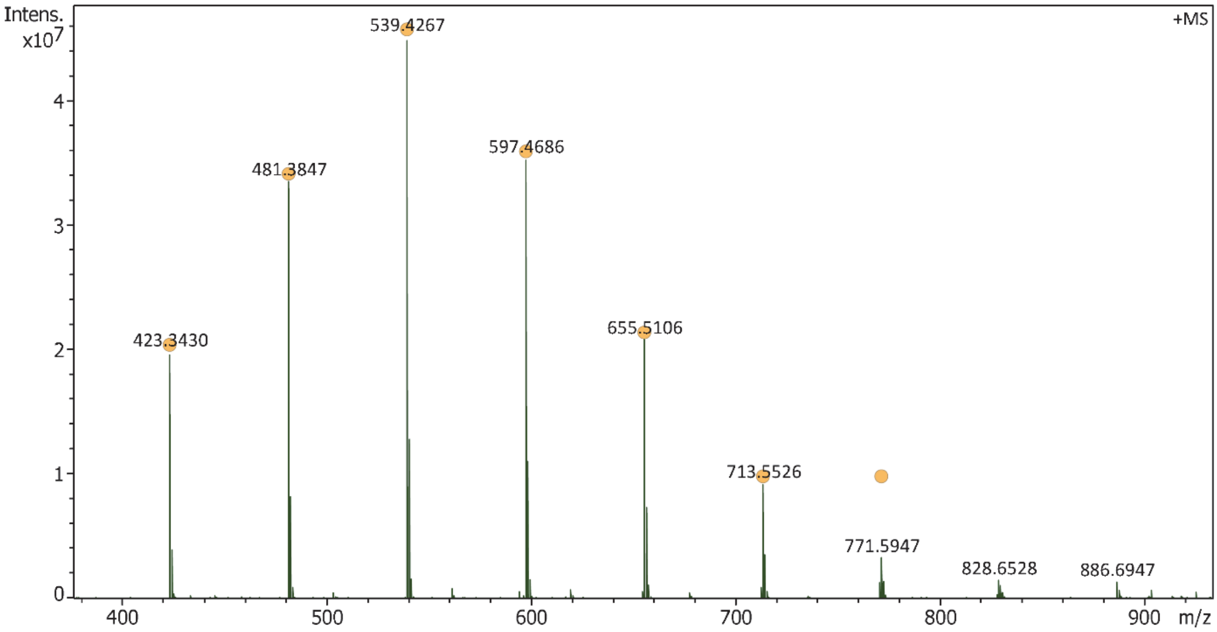

Meas. m/z	Ion Formula	m/z	err [ppm]	mSigma	rdb	e⁻ Conf	N-Rule
365.3011	C18H41N2O5	365.3010	-0.3	12.3	-0.5	even	ok
423.3430	C21H47N2O6	423.3429	-0.2	15.8	-0.5	even	ok
481.3847	C24H53N2O7	481.3847	-0.0	9.7	-0.5	even	ok
539.4267	C27H59N2O8	539.4266	-0.2	15.2	-0.5	even	ok
597.4686	C30H65N2O9	597.4685	-0.2	19.3	-0.5	even	ok
655.5106	C33H71N2O10	655.5103	-0.4	20.3	-0.5	even	ok
713.5526	C36H77N2O11	713.5522	-0.6	23.6	-0.5	even	ok
771.5947	C39H83N2O12	771.5941	-0.9	27.9	-0.5	even	ok

Fig. S12. Positive-ion APFD spectrum of the basic poly(propylene glycol) Jeffamine M-2005. Jeffamine M-2005, a viscous liquid at room temperature, was subjected to APFD analysis using setup C2 (shield at –4.5 kV, cap at –5.0 kV, dry gas at 1.2 l min^{-1} and 100 °C) and without the addition of glycerol matrix. The neat sample delivered an intensive spectrum revealing the series of protonated molecules spaced at $\Delta(m/z)$ = 58.0419 due to the C_3H_6O monomer unit. Formula assignments o ion series are also provided.
The additional ions appearing between the major ion series at tighter spacings from > *m/z* 1000 may be due to various cluster ion compositions and were not further investigated at this stage of the work.

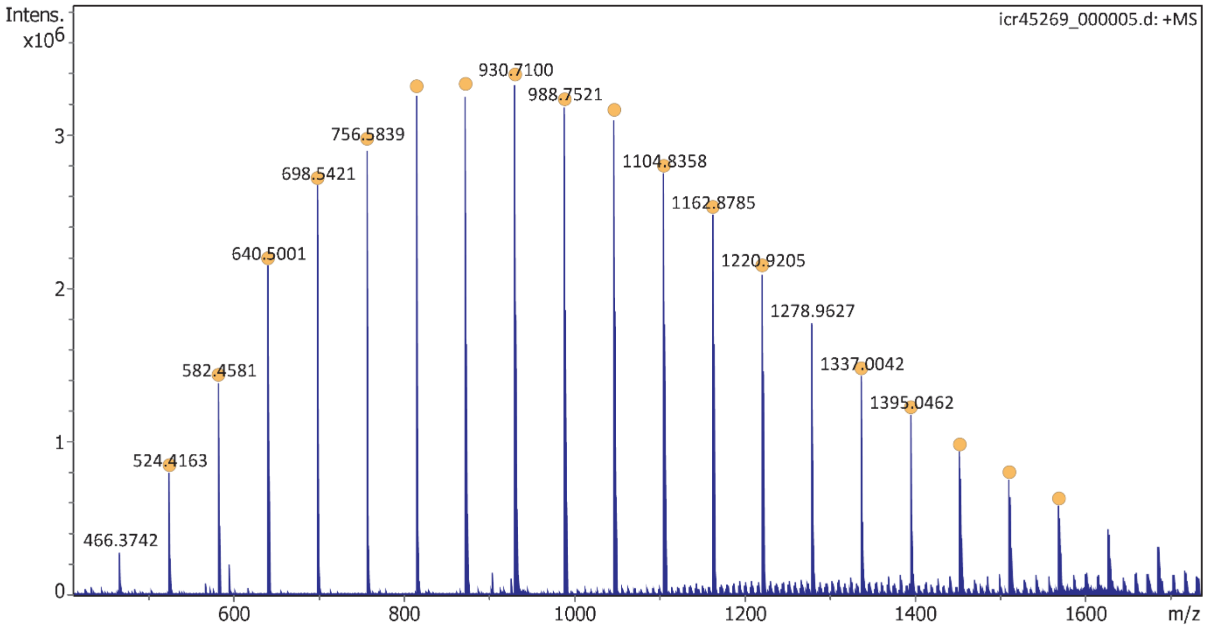

Meas. m/z	Ion Formula	m/z	err [ppm]	mSigma	rdb	e⁻ Conf	N-Rule
466.3742	C24H52NO7	466.3738	-0.7	29.4	-0.5	even	ok
524.4163	C27H58NO8	524.4157	-1.1	5.7	-0.5	even	ok
582.4581	C30H64NO9	582.4576	-0.9	10.0	-0.5	even	ok
640.5001	C33H70NO10	640.4994	-1.1	5.5	-0.5	even	ok
698.5421	C36H76NO11	698.5413	-1.1	7.7	-0.5	even	ok
756.5839	C39H82NO12	756.5832	-1.1	6.8	-0.5	even	ok
814.6260	C42H88NO13	814.6250	-1.2	2.1	-0.5	even	ok
872.6679	C45H94NO14	872.6669	-1.1	4.1	-0.5	even	ok
930.7100	C48H100NO15	930.7087	-1.3	5.0	-0.5	even	ok
988.7521	C51H106NO16	988.7506	-1.5	4.9	-0.5	even	ok
1046.7941	C54H112NO17	1046.7925	-1.5	7.2	-0.5	even	ok
1104.8358	C57H118NO18	1104.8343	-1.3	5.3	-0.5	even	ok
1162.8785	C60H124NO19	1162.8762	-1.9	8.9	0.0	even	ok
1220.9205	C63H130NO20	1220.9181	-2.0	14.9	0.0	even	ok
1337.0042	C69H142NO22	1337.0018	-1.8	14.4	0.0	even	ok
1395.0462	C72H148NO23	1395.0437	-1.9	17.3	0.0	even	ok
1453.0897	C75H154NO24	1453.0855	-2.9	20.5	0.0	even	ok
1511.1323	C78H160NO25	1511.1274	-3.3	24.1	0.0	even	ok
1569.1725	C81H166NO26	1569.1693	-2.1	33.4	0.0	even	ok

Fig. S13. Positive-ion ESI spectrum of the basic poly(propylene glycol) Jeffamine M-2005 where the above solution has been diluted 1:100 in methanol which was infused from a syringe at 8 µl min^{-1}. The intensity distribution of the peaks along the ion series and the *m/z* range covered closely correspond to the APFD spectrum. Formula assignments of the ion series are also provided and are in accordance with the APFD results.

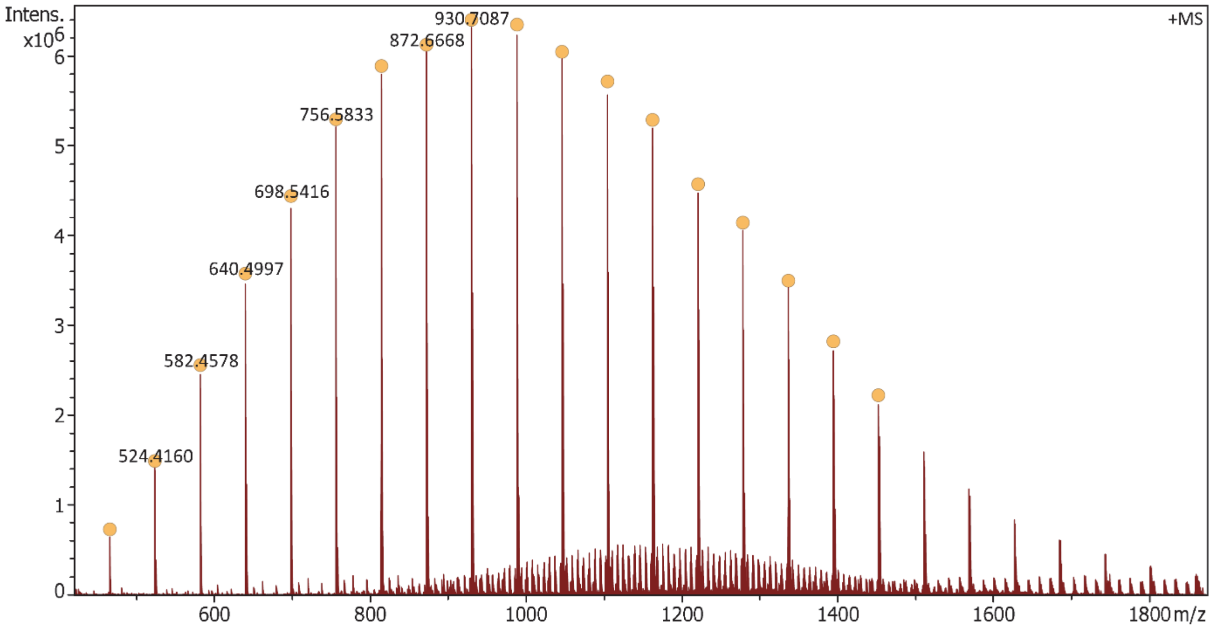

Meas. m/z	Ion Formula	m/z	err [ppm]	mSigma	rdb	e⁻ Conf	N-Rule
408.3322	$C_{21}H_{46}NO_6$	408.3320	-0.5	22.9	-0.5	even	ok
466.3741	$C_{24}H_{52}NO_7$	466.3738	-0.6	9.4	-0.5	even	ok
524.4160	$C_{27}H_{58}NO_8$	524.4157	-0.7	18.4	-0.5	even	ok
582.4578	$C_{30}H_{64}NO_9$	582.4576	-0.4	17.1	-0.5	even	ok
640.4997	$C_{33}H_{70}NO_{10}$	640.4994	-0.4	9.1	-0.5	even	ok
698.5416	$C_{36}H_{76}NO_{11}$	698.5413	-0.4	13.6	-0.5	even	ok
756.5833	$C_{39}H_{82}NO_{12}$	756.5832	-0.2	11.5	-0.5	even	ok
814.6249	$C_{42}H_{88}NO_{13}$	814.6250	0.1	10.8	-0.5	even	ok
872.6668	$C_{45}H_{94}NO_{14}$	872.6669	0.1	5.9	-0.5	even	ok
930.7087	$C_{48}H_{100}NO_{15}$	930.7087	0.1	8.3	-0.5	even	ok
988.7508	$C_{51}H_{106}NO_{16}$	988.7506	-0.2	8.3	-0.5	even	ok
1046.7921	$C_{54}H_{112}NO_{17}$	1046.7925	0.3	15.3	-0.5	even	ok
1104.8335	$C_{57}H_{118}NO_{18}$	1104.8343	0.8	10.0	-0.5	even	ok
1162.8762	$C_{60}H_{124}NO_{19}$	1162.8762	0.0	13.0	-0.5	even	ok
1220.9188	$C_{63}H_{130}NO_{20}$	1220.9181	-0.6	9.5	-0.5	even	ok
1278.9608	$C_{66}H_{136}NO_{21}$	1278.9599	-0.7	17.3	-0.5	even	ok
1336.9998	$C_{69}H_{142}NO_{22}$	1337.0018	1.5	19.7	-0.5	even	ok
1395.0417	$C_{72}H_{148}NO_{23}$	1395.0437	1.4	17.6	-0.5	even	ok
1453.0871	$C_{75}H_{154}NO_{24}$	1453.0855	-1.1	18.4	-0.5	even	ok
